# Assessing differential cell composition in single-cell studies using voomCLR

**DOI:** 10.1093/bioinformatics/btaf637

**Published:** 2025-11-23

**Authors:** Alemu Takele Assefa, Bie Verbist, Koen Van den Berge

**Affiliations:** Statistics and Decision Sciences, Johnson & Johnson, Beerse 2340, Belgium; Statistics and Decision Sciences, Johnson & Johnson, Beerse 2340, Belgium; Statistics and Decision Sciences, Johnson & Johnson, Beerse 2340, Belgium

## Abstract

**Motivation:**

In single-cell studies, a common question is whether there is a change in cell composition between conditions. While ideally, one needs absolute cell counts (number of cells per volumetric unit in a sample) to address these questions, current experimentation typically obtains cell counts that only carry relative information. Therefore, one should account for the compositional nature of cell count data in the statistical analysis. While recently developed methods address compositionality using compositional transformations together with a bias correction, they do not account for the uncertainty involved in estimation of the bias term, nor do they accommodate the mean–variance structure of the counts.

**Results:**

Here, we introduce a statistical method, voomCLR, for assessing differences in cell composition between conditions incorporating both uncertainty on the bias term as well as acknowledging the mean–variance structure of the transformed data, by leveraging developments from the differential gene expression literature. We demonstrate the performances of voomCLR, illustrate the benefit of all components, and compare the methodology to the state-of-the-art on simulated and real single-cell gene expression datasets.

**Availability and implementation:**

voomCLR software is available as an open-source R package on GitHub at https://github.com/johnsonandjohnson/voomCLR.

## 1 Introduction

High-dimensional biological single-cell datasets, where multiple features are measured per single cell, are ubiquitous in modern biology, e.g. flow cytometry and single-cell RNA-sequencing (scRNA-seq) experiments. Usually, the features measured at cell-level (e.g. gene expression or chromatin accessibility) are used to categorize cells into cell types and/or cell states (Amezquita et al. 2020). Conditional on such a grouping of cells, a common task concerns the estimation of cell population composition and contrasting cell population abundances between (any number of) conditions; i.e. a difference in the cell type/state composition (Frishberg *et al.* 2019, Phipson *et al.* 2022).

Ideally, one would like to observe the absolute counts for each cell population (e.g. number of cells for each population per unit volume of blood in a patient). However, the observed data from a sequencing experiment only capture relative abundance information as the number of cells observed for a particular sample does not reflect the total number of cells in the sample (Quinn *et al.* 2018) but is rather constrained to an arbitrary sum. The field of compositional data analysis is concerned with performing inference on the unobserved absolute cell counts using the observed data which only contains relative information (Aitchison 1982).

Many methodologies are described in the literature to analyze changes in cell composition. First, several approaches exist for unsupervised differential abundance (DA) analysis, which are unsupervised in the sense that no *a priori* known grouping of the cells is provided and instead rely on approaches like *k*-nearest neighbor graphs to categorize cells (Zhao *et al.* 2021, Dann *et al.* 2022). Here, we will instead focus on the scenario where a biologically relevant cell grouping has been provided and is followed by a DA analysis. In what follows, we refer to a relevant grouping of cells as a ‘cell type’ or ‘cell population’, but note that this is equally applicable for any other biologically relevant disjoint cell grouping. A common though naive approach of DA analysis fits a negative binomial generalized linear model (NB-GLM) for each cell type, using the cell type counts as a response (Domingo *et al.* 2023, Maity and Teschendorff 2023). By incorporating the total number of cells per sample as offset, one models the relative abundance of each cell type. This approach ignores the compositional nature of the data, leading to inflated false positive rates (Zhou *et al.* 2022). Indeed, compositional cell population count data are negatively correlated between populations, implying that an increase in abundance of one population will lead to a decrease of other populations, and ignoring these effects leads to an increased risk of identifying false positives. An improvement to this approach is to normalize the total cell count of each sample, and use the normalized total as offset. This is often done by using tools originally developed for bulk RNA-sequencing differential expression analysis (Weber *et al.* 2019), like edgeR and DESeq2 (Robinson *et al.* 2010, Love *et al.* 2014). Other methods effectively take into account the compositional nature of the data by using appropriate distributional assumptions. DCATS uses a Beta-Binomial regression framework for each cell type to infer differences in cell composition, where the dispersion parameter is estimated jointly across all cell types (Lin *et al.* 2023). sccomp similarly relies on a Beta-Binomial model and allows for testing differential composition and variability between the groups (Mangiola *et al.* 2023). scCODA adopts a Bayesian multivariate regression framework based on the Dirichlet-Multinomial distribution, a generalization of the Beta-Binomial distribution (Büttner *et al.* 2021). Another class of methods instead transform the cell type counts; propeller adopts a logit or arscin square root transformation and uses linear models post-transformation (Phipson *et al.* 2022). Alternatively, compositional transformations, a class of transformations originally proposed by Aitchison (1982, 1986), are often used. They aim at transforming the data out of the simplex space, and mapping them into the real space, making measures like Euclidean distances and least squares meaningful (Quinn *et al.* 2018). LinDA adopts a compositional transformation called the centered log-ratio (CLR) and also uses linear models downstream (Zhou *et al.* 2022). The authors then show that the estimated effect sizes are biased with respect to the effect sizes one would obtain based on the absolute abundances, and propose a bias correction approach based on the mode of the effect size across cell types. Inference then occurs on the bias-corrected effect size, relying on the standard error pre-correction. While the approach was developed for DA testing in microbiome data, we here show that it works very well for assessing changes in cell type composition, too.

In this article, we compose a method for DA analysis through leveraging advances of the recent literature and filling missing methodological gaps. We follow the rationale of the LinDA methodology by using a CLR transformation, fitting linear models, and adopting bias correction on the effect sizes. We extend this approach in two major directions. First, we account for the uncertainty involved in estimating the bias correction term by adopting a bootstrapping approach. Second, we show that the cell type counts are still heteroscedastic post-transformation, and account for the counts’ mean–variance structure using heteroscedasticity weights by building on the limma-voom framework. This has the additional advantage that we can adopt their empirical Bayes approach for shrinking linear model residual variances. After discussing these extensions in Sections 3.1 and 3.2, we evaluate our approach, called voomCLR, alongside the state-of-the-art in both simulation studies (Section 3.3) and real data analyses (Sections 3.4 and 3.5).

## 2 Materials and methods

### 2.1 Bias correction when modeling transformed counts

Consider a log-linear model on absolute abundances $X_{ip}$ for each population *p* ∈ {1, …, *P*} in sample *i* ∈ {1, …, n}


(1) $$\text{log}\;X_{ip}=C_{i}\alpha_{p}+\epsilon_{ip},$$

with $\alpha_{p}$ the (k+1)×1 vector of regression coefficients and $C_{i}$ being the row corresponding to sample *i* of the n×(k+1) design matrix C, used for representing the experimental covariates taken into account. In a DA analysis, we are interested in testing the null hypothesis that (a linear combination of) $\alpha_{p}$, say $\alpha_{jp}$, is equal to zero, i.e. $H_{0}:\alpha_{jp}=0$ for every p∈{1,…,P}.We usually do not have access to the absolute abundances $X_{ip}$ but instead must work with the observed cell type counts $Y_{ip}$. Since these data only contain relative abundance information, one may consider a CLR transformation, i.e.


(2) $$Z_{ip}=\text{log}\;\frac{Y_{ip}}{{\tilde{Y}}_{i}}\;\text{with}\;{\tilde{Y}}_{i}={\left(\prod_{p=1}^{P}Y_{ip}\right)}^{1/P}.$$

We could similarly fit a linear model on the transformed data,


(3) $$Z_{ip}=\beta_{0p}+\sum_{j=1}^{k}\beta_{jp}C_{ij}+e_{ip}$$

However, regression coefficients β from this linear model are biased with respect to the coefficients from Equation (1) (Zhou *et al.* 2022). Indeed, Zhou *et al.* (2022) show that $\beta_{jp}=\alpha_{jp}-{\bar{\alpha }}_{j}$, with ${\bar{\alpha }}_{j}=\frac{1}{P}\sum_{p=1}^{P}\alpha_{jp}$. Since $\alpha_{jp}$ and ${\bar{\alpha }}_{j}$ are unidentifiable given an estimate for $\alpha_{jp}-{\bar{\alpha }}_{j}$, Zhou *et al.* (2022) make the assumption that most $\alpha_{jp}=0$, or more precisely that the mode of the distribution of $\alpha_{jp}$ across populations, equals zero. Given this assumption, they estimate ${\bar{\alpha }}_{j}$ by shifting the distribution of our estimates of $\alpha_{jp}-{\bar{\alpha }}_{j}$ such that it has a mode at zero. The shift is our estimate for ${\bar{\alpha }}_{j}$, hence providing us with the bias correction term. Concretely, we calculate the bias-corrected coefficient


(4) $${\tilde{\beta }}_{jp}=\beta_{jp}-{\overset{ˇ}{\beta }}_{j},$$

with ${\overset{ˇ}{\beta }}_{j}$ the mode of the $\beta_{jp}$ parameters from Model (3), calculated across cell types.

### 2.2 Accounting for the uncertainty of estimating the bias correction term

The variance on ${\tilde{\beta }}_{jp}$ is


(5) $$\mathrm{Var}({\tilde{\beta }}_{jp})=\mathrm{Var}(\beta_{jp})+\mathrm{Var}({\overset{ˇ}{\beta }}_{j})-2Cov(\beta_{jp},{\overset{ˇ}{\beta }}_{j}).$$

Zhou *et al.* (2022) argue that as the number of samples and the number of features tend to infinity, $\mathrm{Var}(\beta_{jp})$ dominates $\mathrm{Var}({\overset{ˇ}{\beta }}_{j})$ and $\mathrm{Cov}(\beta_{jp},{\overset{ˇ}{\beta }}_{j})$ under mild conditions, and therefore rely only on $\mathrm{Var}(\beta_{jp})$ for statistical inference on ${\tilde{\beta }}_{jp}$. However, in the context of modeling cell type composition this is an unrealistic argument as the number of cell types (features) is usually limited. We therefore attempt to provide a closer approximation to the variance of the bias-corrected parameter by accounting for additional uncertainty involved in estimating the bias correction term, $\mathrm{Var}({\overset{ˇ}{\beta }}_{j})$. For each (linear combination of) parameter(s) of interest, say $L^{T}\beta_{p}$, with L a k×1 vector specifying the linear combination of interest, a non-parametric bootstrap procedure (Efron 1979) is adopted by resampling $\beta_{jp}$ across *p*, with replacement, and recalculating $L^{T}\beta_{p}$ as needed. By default, B=4000 resamples are taken and, for each j∈{0,…,k}, the dimension of the bootstrapped $\beta_{j}{}^{*}$ is the same as for the original vector $\beta_{j}$, i.e. P×1. For each bootstrap sample and each (linear combination of) coefficient(s), we calculate the mode ${\overset{ˇ}{\beta }}_{jb}$ and we approximate the variance of the bias term by


(6) $$\hat{\mathrm{Var}({\overset{ˇ}{\beta }}_{j})}=\frac{1}{B-1}\sum_{b=1}^{B}{\left({\overset{ˇ}{\beta }}_{jb}-{\bar{\overset{ˇ}{\beta }}}_{j}\right)}^{2},$$

with ${\bar{\overset{ˇ}{\beta }}}_{j}=\frac{1}{B}\sum_{b=1}^{B}{\overset{ˇ}{\beta }}_{jb}$. If interest lies in testing the null hypothesis that $H_{0}:\beta_{jp}=0$, then this would correspond to


(7) $$T_{jp}=\frac{{\hat{\tilde{\beta }}}_{jp}}{\sqrt{\hat{\mathrm{Var}({\hat{\beta }}_{jp})}+\hat{\mathrm{Var}({\overset{ˇ}{\beta }}_{j})}}},$$

where $\hat{\mathrm{Var}({\hat{\beta }}_{jp})}$ is the estimated moderated variance on ${\hat{\beta }}_{jp}$ from limma (Smyth 2004).

In Supplementary Methods, available as supplementary data at *Bioinformatics* online, we describe an analogous parametric bootstrap procedure, which additionally allows to take into account the covariance term in Equation (5).

### 2.3 Simulation frameworks and case study analyses

Details on simulation frameworks, case study analyses, and details on implementation of other methods can be found in Supplementary Methods, available as supplementary data at *Bioinformatics* online.

### 2.4 Availability of code and data

Code to reproduce the simulations and case studies is available from GitHub at https://github.com/koenvandenberge/voomCLRPaper. The software voomCLR is available at https://github.com/johnsonandjohnson/voomCLR.

## 3 Results

### 3.1 Compositional transformations lead to biased parameter estimates

The information content in compositional data is in the relative abundance of the features being measured (Aitchison 1982). It is, e.g. insensible to analyze the cell counts of one particular cell type, while ignoring all other cell types, as the counts for the individual cell type contain no relevant information for analyzing compositional differences. This has inspired a range of compositional transformations that transform compositional data out of the simplex space, often through calculating log-ratios of the counts, after which standard multivariate techniques may be used for downstream analysis (Aitchison 1982). The denominator of the log ratio is usually either (i) the count of another feature (i.e. the count of another cell type) from the same sample, in which case the transformation is called the additive log-ratio (ALR); (ii) the geometric mean of all counts from that sample, referred to as the CLR transformation (Aitchison 1986). Various extensions to these transformations have been developed more recently, e.g. Silverman *et al.* (2017).

Letting $Y_{ip}$ denote the observed cell type count of population *p* in sample *i*, these transformations may be written as


(8) $$\text{ALR:}\;\;\text{log}\;\frac{Y_{ip}}{Y_{iq}}\;\text{for}\;p\neq q\text{CLR:}\;Z_{ip}=\text{log}\;\frac{Y_{ip}}{{\tilde{Y}}_{i}}\;\text{with}\;{\tilde{Y}}_{i}={\left(\prod_{p=1}^{P}Y_{ip}\right)}^{1/P}.$$

**Figure 1.**
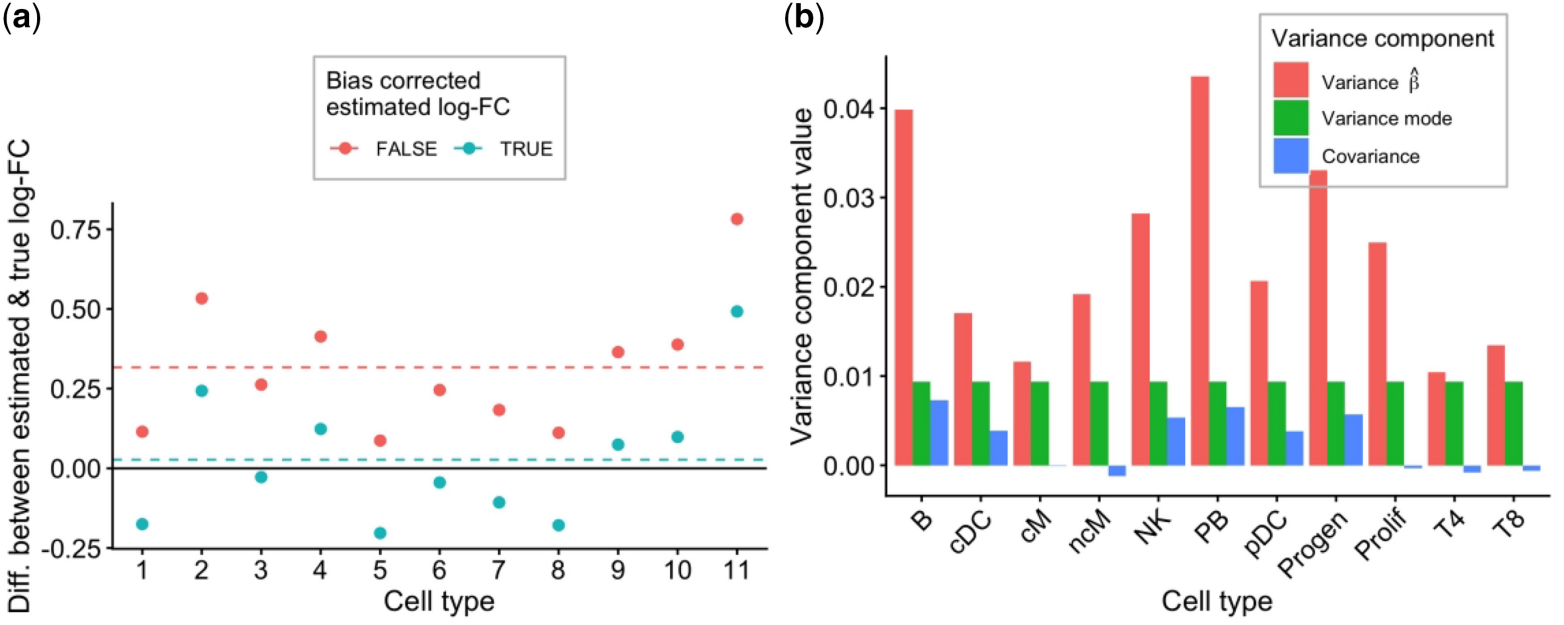
Bias correction and associated uncertainty. (a) Effect sizes between two groups using our parametric Dirichlet-Multinomial simulation framework. The data points represent difference of estimated minus true effect sizes (*y*-axis) for each cell type (*x*-axis). The red data points are from a voomCLR analysis without applying bias correction, and can be seen to be biased on average (red dashed line). The green data points are from a voomCLR analysis where bias correction was applied and are unbiased on average (green dashed line). (b) Variance components for the bias-corrected effect sizes. The red bar represents the estimated variance on the estimated effect size as obtained from the weighted linear model in voomCLR. The green bar is the estimated variance on the bias correction term, which is the same for all cell types. The blue bar represents the estimated covariance between the two. The latter two values were estimated using our parametric bootstrap procedure.

Let $X_{ip}$ denote the (unobserved) absolute abundance of each population *p* in sample *i*, e.g. the number of T-cells in a person’s blood per volumetric unit. In the context of DA analysis we are interested in testing the null hypothesis that the log-fold-change on the expected absolute abundances is equal to zero. However, when using the (observed) CLR-transformed counts $Z_{ip}$ as a response variable in a linear model it has been shown that the obtained log-fold-changes are biased with respect to the log-fold-changes on the absolute counts $X_{ip}$ (Zhou *et al.* 2022) (see Section 2 for detailed derivations). We indeed confirm the bias using a simulation study based on the multinomial distribution (Fig. 1a). In the microbiome literature, Zhou *et al.* (2022) proposed a bias correction which we also found to alleviate the bias in our context (Fig. 1a). Assuming that the majority of populations are not DA, the correction amounts to subtracting the mode of the regression coefficients across populations, a computationally tractable approach that we will be leveraging in subsequent sections.

Following bias correction, the LinDA method from Zhou *et al.* (2022) performs inference on the bias-corrected effect sizes, assuming that the bias term was known rather than estimated. Zhou *et al.* (2022) argue that as the number of samples and the number of features tend to infinity, the uncertainty on the bias term becomes negligible as compared to the uncertainty on the (uncorrected) effect size. However, in the context of modeling cell type composition this is an unrealistic assumption as the number of cell types (features) is usually limited. Figure 1b shows that the variance on the bias correction can constitute a substantial part of the total variance (see Section 2 for its formulation). We therefore approximate the variance of the bias-corrected parameter by developing a bootstrap procedure to account for additional uncertainty involved in estimating the bias correction term, and incorporate it in downstream statistical inference. As can be seen in our simulation studies, this aids substantially in controlling the number of false positive results.

### 3.2 Compositional transformations do not stabilize variances

**Figure 2.**
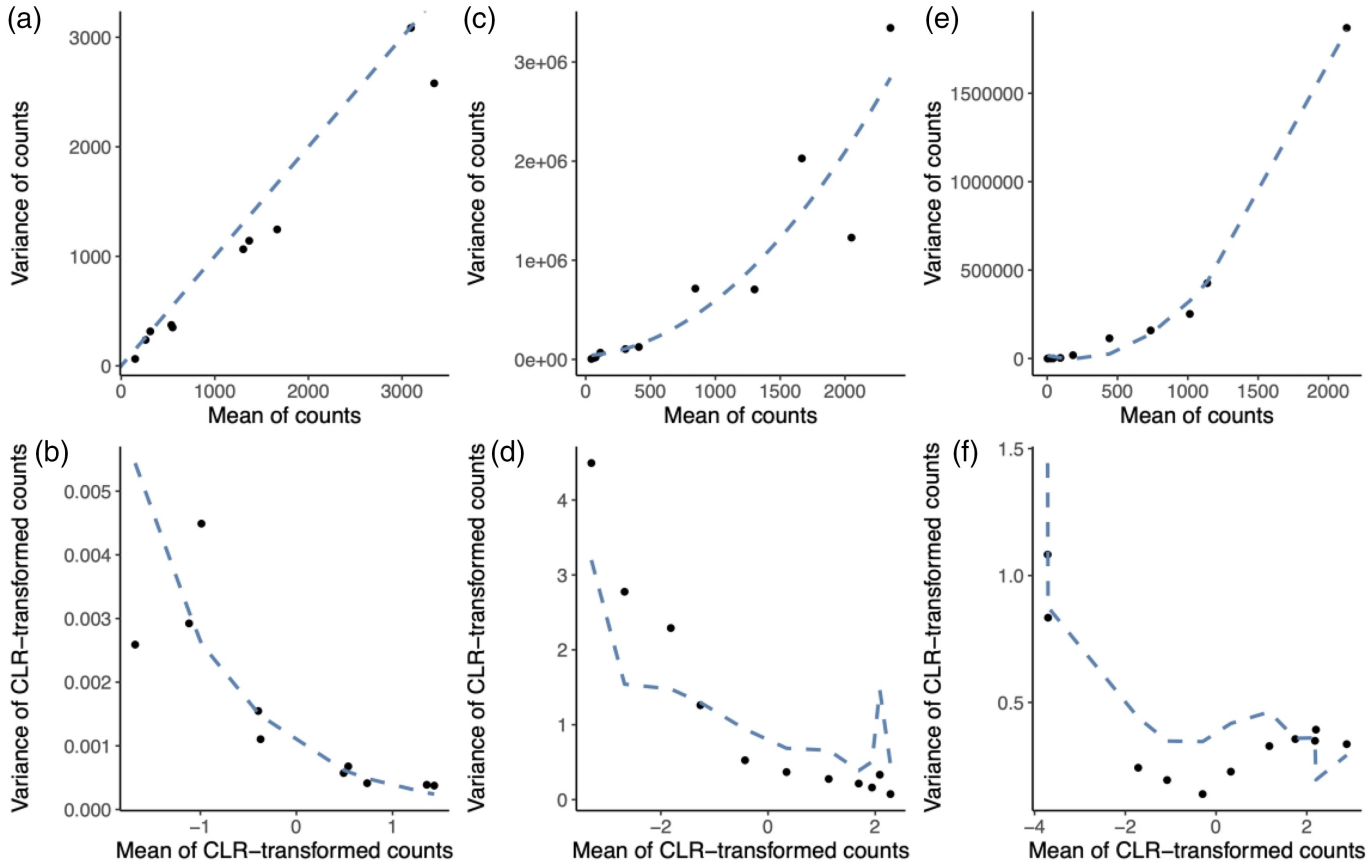
Heteroscedasticity for simulated and real data. (a–b) Simulation based on Multinomial distribution: Panel (a) shows the empirical mean–variance relationship of the counts for each cell type, with the dashed line being the identity line. Panel (b) shows the empirical mean–variance relationship of the CLR-transformed cell type counts, with the dashed line being the analytically calculated variance using the delta method based on the Poisson distribution. (c–d) Simulation based on a Dirichlet-Multinomial distribution: Panel (c) shows the empirical mean–variance relationship of the counts for each cell type, with the dashed line being the estimated mean–variance trend. Panel (d) shows the empirical mean–variance relationship of the CLR-transformed cell type counts, with the dashed line being the analytically calculated variance using the delta method based on the negative binomial distribution. (e–f) Lupus dataset, upon selecting the healthy patients from processing cohort 1: Panel (e) shows the empirical mean–variance relationship of the counts for each cell type, with the dashed line being the estimated mean–variance trend. Panel (f) shows the empirical mean–variance relationship of the CLR-transformed cell type counts, with the dashed line being the analytically calculated variance using the delta method based on the negative binomial distribution. Note that the trend in panels (d) and (f) are not smooth since they rely on a cell type specific dispersion estimate.

The transformed counts $Z_{ip}$ may be used downstream as the response variable in a linear model for statistical inference (Zhou *et al.* 2022, Hu and Satten 2023). However, since the untransformed data constitute counts, they have a mean–variance relationship, which is not taken into account when using a vanilla linear model on the transformed data. We explore the mean–variance relationship using both a null simulation study assuming a Multinomial distribution for $Y_{ip}$ as well as real data. Figure 2a shows that, for the Multinomial simulation, the mean–variance relationship for the cell population counts across samples can be approximated by a Poisson law (see Section 2). After CLR transformation, the variance is not independent of the mean, and indeed is a decreasing function of the mean CLR-value (Fig. 2b). We see similar patterns using our parametric Dirichlet-Multinomial simulation framework (Fig. 2c and d), as well as using real scRNA-seq data (Fig. 2e and f). Using the first-order delta method (Doob 1935, Dorfman 1938) (see Supplementary Methods, available as supplementary data at *Bioinformatics* online) one can approximate the variance of the transformed data to theoretically show it is still a function of the mean, i.e. the compositional transformations are not variance-stabilizing:


(9) $$\text{If}\;Y_{ip}∼\mathrm{Poi}(\lambda_{ip}),\;\mathrm{Var}(Z_{ip})\approx {\left(\frac{P-1}{P}\right)}^{2}\frac{1}{\lambda_{ip}}.$$

(10) $$\text{If}\;Y_{ip}∼NB(\mu_{ip},\phi_{p}),\mathrm{Var}(Z_{ip})\approx {\left(\frac{P-1}{P}\right)}^{2}\left(\frac{1}{\mu_{ip}}+\phi_{p}\right).$$

Law *et al.* (2014) previously transformed RNA-seq counts using a log-counts-per-million transformation. They addressed the heteroscedasticity of these transformed counts by estimating an empirical mean–variance trend across all genes and leverage this trend to incorporate the mean–variance relationship through observation-level weighting in a linear model. Here, we instead use the heteroscedasticity weights upon CLR transformation of the cell type abundance counts and estimate the trend across cell populations (instead of genes). By further leveraging the software implementation, we also gain by adopting the empirical Bayes shrinkage of linear model residual variances, across cell types (Smyth 2004). This helps especially in small sample size settings. However, in contrast to RNA-seq data where one observes thousands of genes, some datasets may only consist of a limited number of cell populations, rendering the empirical estimation of the mean–variance trend uncertain. We therefore also provide the option to derive the weights analytically, using the delta method (see Supplementary Methods, available as supplementary data at *Bioinformatics* online).

### 3.3 Simulation studies

**Figure 3.**
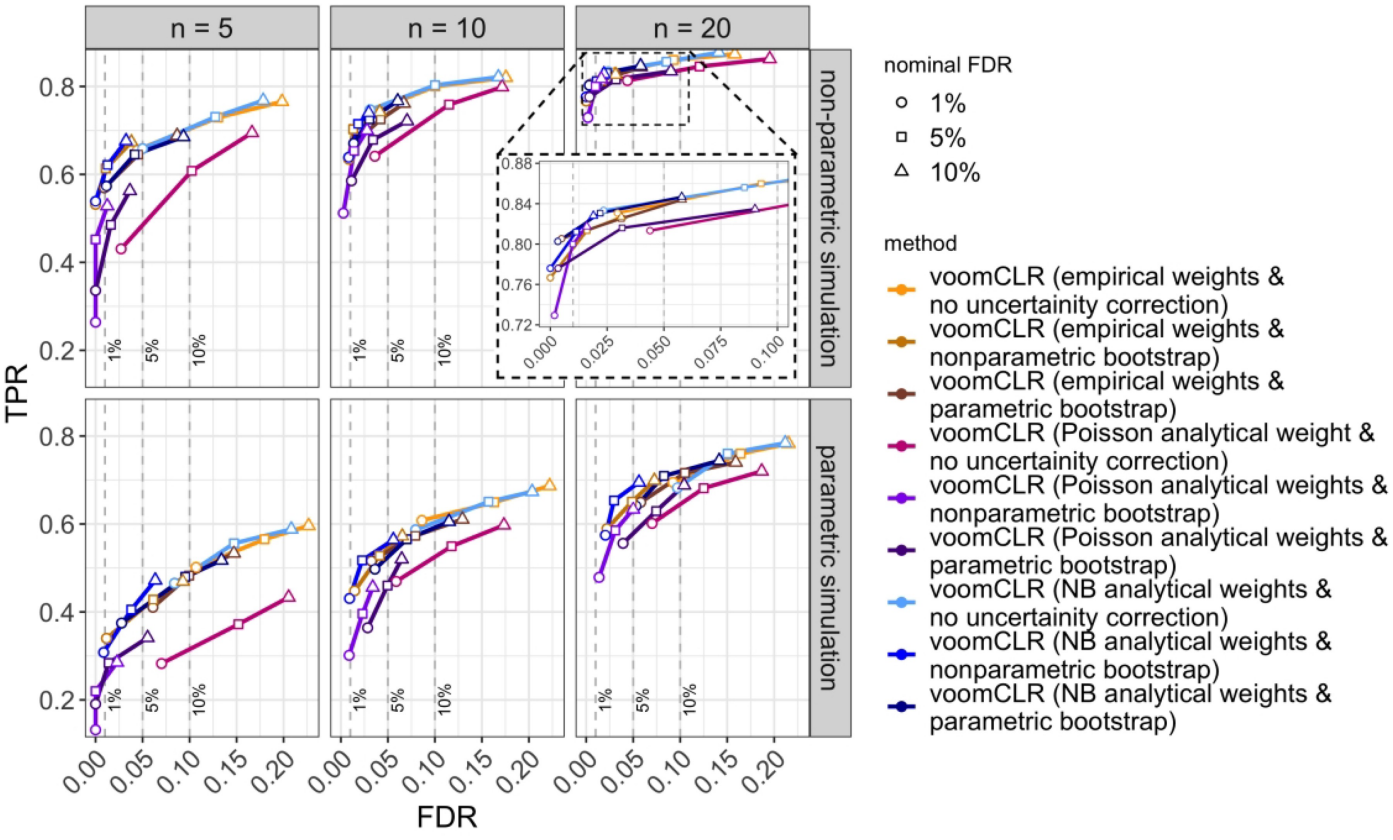
FDR-TPR performance curves of nine different voomCLR configurations in two simulation paradigms. The curves indicate the performance of the methods for testing differential abundance in simulated compositional cell count data. Performance metrics (FDR and TPR) are calculated at 1%, 5%, and 10% nominal FDR levels for each simulation setting. Two simulation strategies are used: (i) non-parametric simulation based on recycling real cell abundance data (using the lupus dataset from Perez *et al.* (2022)), and (ii) parametric compositional cell count data simulation using a Dirichlet-Multinomial distribution. Reported metrics are averages from 250 simulation runs for each simulation scenario. Each simulated dataset consists of two independent groups of samples for 11 cell populations. Data are simulated with a sample size of 5, 10, and 20 per group and with a medium level of variability between samples (Dirichlet parameters scaling factor γ=1). An interactive version of this figure can be found on our GitHub repository. NB = negative binomial.

**Figure 4.**
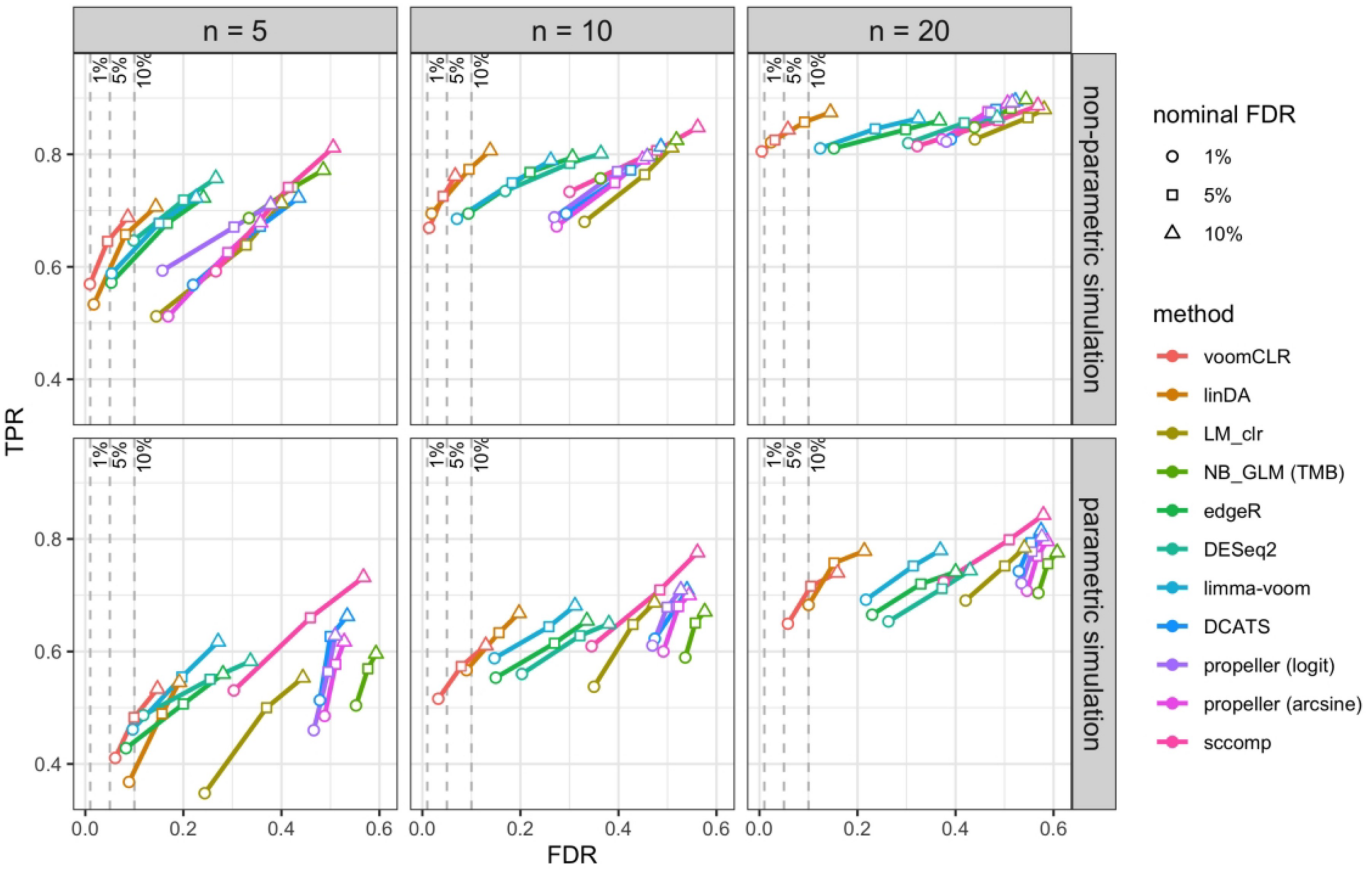
FDR-TPR performance curves of 10 methods from two simulation paradigms. The curves indicate the performance of the methods for testing differential abundance in simulated compositional cell count data. Two simulation strategies are used: (i) non-parametric simulation based on recycling real cell abundance data (using the lupus dataset from Perez *et al.* (2022)), and (ii) parametric compositional cell count data simulation using a Dirichlet-Multinomial distribution. Performance metrics (FDR and TPR) are calculated at 1%, 5%, and 10% nominal FDR levels for each simulation setting. Reported metrics are averages from 250 simulation runs for each simulation scenario. Each simulated dataset consists of two independent groups of samples for 11 cell populations. Data are simulated with a sample size of 5, 10, and 20 per group and with a medium level of variability between samples (Dirichlet parameters scaling factor γ=1).

**Figure 5.**
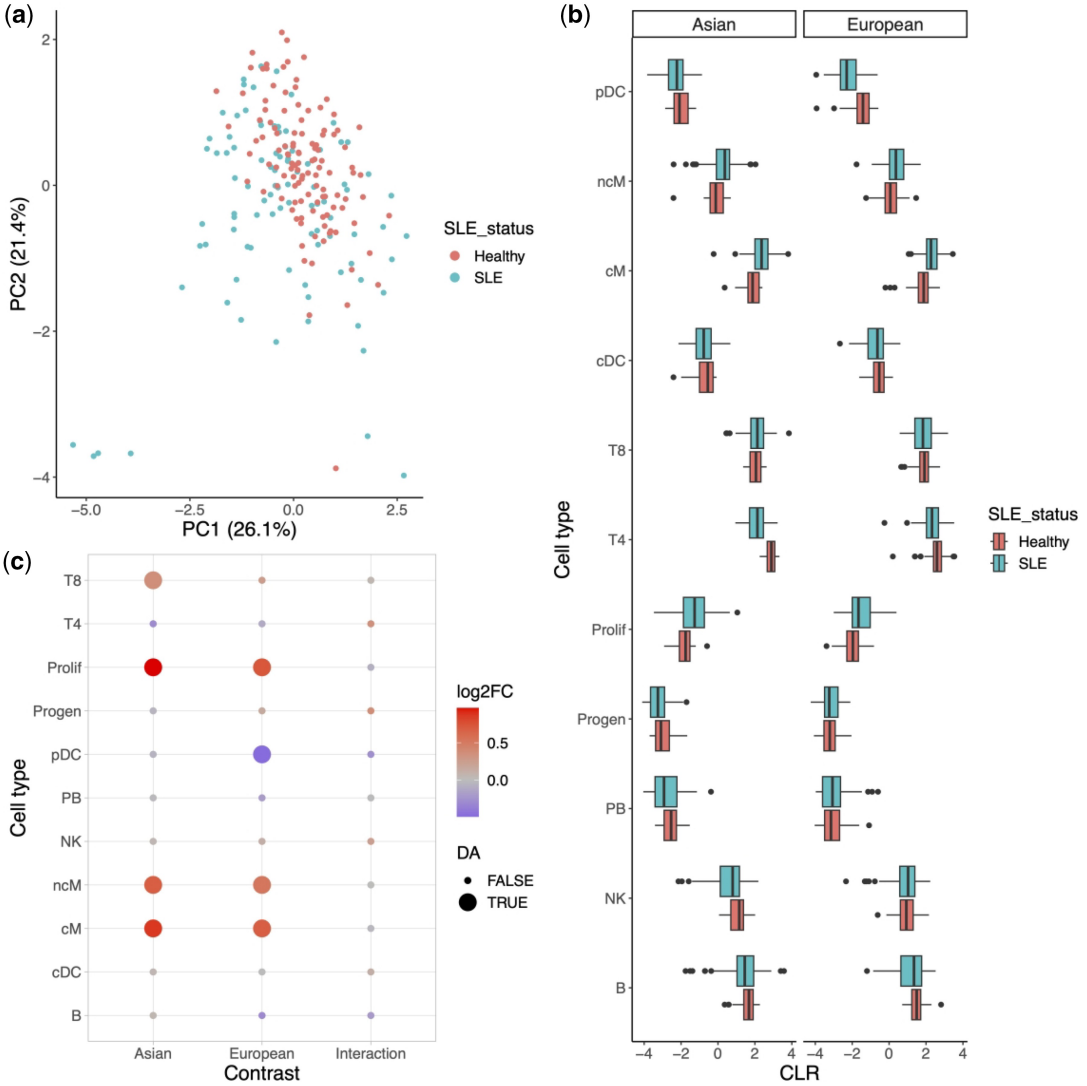
Lupus case study. (a) Scatterplot of first two principal components of Aitchison’s distance PCA for all samples. Each sample is colored by disease status. (b) Boxplots of CLR-transformed cell type abundances, for both the Asian and European ancestries. (c) Heatmap showing results of voomCLR for all cell types (*y*-axis). Each point is colored according to the log2 fold-change, and point size denotes whether the FDR-adjusted *P*-value is below the 10% level.

In this section, we will first evaluate the performance of voomCLR with respect to different options of accounting for heteroscedasticity and uncertainty of the bias correction. Next, we compare voomCLR to popular and state-of-the-art statistical methodology to assess differences in cell type composition. The evaluation centers around two key characteristics, the false discovery rate (FDR) and the sensitivity (also known as the true positive rate or TPR). Both of these characteristics are computed at a specified nominal FDR level. We employed two approaches to simulate realistic cell count data: (i) a non-parametric framework in which signal is introduced in a real dataset; and (ii) a parametric simulation in which cell counts are generated from a Dirichlet-Multinomial distribution. We limited the simulations to a two-group comparison of independent replicates. The details of these simulation methods can be found in the Supplementary Methods section, available as supplementary data at *Bioinformatics* online. Figs 1–5, available as supplementary data at *Bioinformatics* online, visualize the comparison between a real and a representative simulated dataset, showing simulated data captures essential characteristics of real data.

#### 3.3.1 Performance of voomCLR

Figure 3 shows the performance of voomCLR across nine different configurations of the method. These include three options to accommodate the estimation uncertainty of the bias correction factor (none, non-parametric bootstrap, or parametric bootstrap) combined with three approaches to estimate the observation-level heteroscedasticity weights (empirical weights or analytically calculated weights based on either a Poisson or negative binomial distribution). We compare these configurations in detail in Supplementary Results as well as Figs 6–9, available as supplementary data at *Bioinformatics* online. In general, accounting for the uncertainty of the bias correction improves FDR control. The non-parametric bootstrap procedure is conservative in terms of FDR control and therefore comes with an associated cost of reduced sensitivity. On the other hand, the parametric bootstrap is moderately liberal and is associated with higher sensitivity. For medium to high numbers of cell types, the choice between empirical and negative binomial-based analytical weights does not have a significant impact, while for low numbers of cell types the empirical trend becomes too uncertain and the negative binomial-based analytical weights are preferred. In general, the Poisson-based weights underperform.

Based on this performance evaluation, voomCLR with NB analytical weights and parametric bootstrap is chosen as a default method in the subsequent performance evaluations presented in the article; we will use the shorter name voomCLR to refer to this configuration.

#### 3.3.2 Performance of voomCLR compared to other methods

The simulation study further allows evaluating the performance of voomCLR compared to other popular methods for DA analysis. The methods included in the evaluations can be categorized into three groups. First, methods that apply linear models on CLR-transformed cell counts. Under this category, we have voomCLR, LinDA (Zhou *et al.* 2022), and as a baseline a vanilla linear model using the CLR-transformed counts as response (LM_CLR)—which makes inference based on the estimated effect sizes with neither compositional bias nor heteroscedasticity correction. In the second category, we have methods developed for differential expression analysis of digital gene expression data. The methods under this category are edgeR (Robinson *et al.* 2010), DESeq2 (Love *et al.* 2014), and limma-voom (Law *et al.* 2014). edgeR and DESeq2 employ a negative binomial generalized linear model (NB-GLM) framework for testing DA. limma-voom fits linear models on log-transformed counts per millions (CPM). It shares various techniques, such as normalization of total cell counts and moderated tests, with edgeR and DESeq2. As a baseline for the second category, we have included ordinary negative binomial GLMs (NB_GLM (TMB)) which use the logarithm of the total cell count per sample as an offset, fitted using the glmmmTMB R package (Brooks *et al.* 2017). The third category includes methods specifically developed for testing DA in compositional cell population data often originating from single-cell RNA-seq data. These include propeller (with logit or arcsine transformation of cell counts) (Phipson *et al.* 2022), DCATS (Lin *et al.* 2023), and sccomp (Mangiola *et al.* 2023). A brief description of all these methods and implementation notes can be found in the Supplementary Methods section, available as supplementary data at Bioinformatics online.

The results presented in Fig. 4 compare the 10 methods discussed above based on a simulation setting with 11 cell populations (P=11) at three different sample sizes (*n* = 5, 10, and 20 in each group), similar to Fig. 3. Notably, two methods—voomCLR and LinDA—stand out with respect to FDR control compared to all the other methods. In contrast, the linear model applied to CLR-transformed counts without bias correction, heteroscedasticity correction, or empirical Bayes shrinkage performs poorly in terms of both FDR control and TPR.

In particular, the results in Fig. 4 indicate that voomCLR generally outperforms LinDA especially when the sample size is low (n≤10) or when there is medium to large variability between samples (Fig. 10, available as supplementary data at *Bioinformatics* online). voomCLR achieves this while maintaining a comparable TPR for detecting truly differentially abundant cell populations. Methods developed for differential gene expression analysis, edgeR, DESeq2, and limma-voom demonstrated improved performance compared to that of a vanilla NB-GLM, which can be explained by the total count normalization procedure these tools adopt (Fig. 11, available as supplementary data at *Bioinformatics* online). Nevertheless, for a typical number of cell populations, i.e. between 10 and 30, edgeR, DESeq2, and limma-voom underperform compared to voomCLR in terms of FDR control whereas, in simulated data with more than 50 cell populations, these methods perform relatively well and comparable to voomCLR (Fig. 12, available as supplementary data at *Bioinformatics* online). DCATS, sccomp, and propeller generally did not perform well in both simulation settings (Fig. 4 and Figs 10 and 12, available as supplementary data at *Bioinformatics* online). Results from the non-parametric simulation study based on the breast atlas case study dataset, and a comparison between this simulated dataset and the one based on the lupus case study dataset, are shown in Figs 13 and 14, available as supplementary data at Bioinformatics online.

In summary, voomCLR is a robust, powerful method that, across a broad range of scenarios and simulation frameworks, dominates other methods.

### 3.4 Lupus case study

Perez *et al.* (2022) perform scRNA-seq on 355 human blood samples from 261 individuals, corresponding to 99 healthy controls and 162 systemic lupus erythematosus (SLE), hereafter also referred to as ‘lupus’, patients. Following the analysis from the original paper, we here focus on samples from humans with Asian and European ancestry. After quality control and initial filtering, more than 1.2 million cells remained, which were assigned to 11 cell types (Perez *et al.* 2022). We perform exploratory data exploration on the cell composition data consistent with compositional data analysis by using a principal component analysis based on Aitchison’s distance. While the healthy samples are more tightly grouped as compared to SLE samples (Fig. 5a), both the groups overlap. We find no known technical variation to be associated with the variance captured in the reduced dimension plot (Fig. 15, available as supplementary data at *Bioinformatics* online). The CLR-transformed data are shown in Fig. 5b. We model the data using voomCLR accounting for processing cohort as a covariate, and also include an interaction between lupus status and ancestry, as in the original paper the lupus status had been assessed for samples from Asian and European ancestry separately. We account for replicate sequencing of some individuals through the duplicateCorrelation feature from limma. Given the superior performance of the parametric bootstrap in our non-parametric simulation study based on this dataset, we account for uncertainty of the bias correction using the parametric bootstrap. Statistical inference is performed on a 10% nominal FDR level, testing the lupus disease effect within Asian and European ancestries, as well as the interaction between lupus disease status and ancestry. In doing so, we confirm the finding of the original paper that, for both ancestries, the average abundance of cM and proliferating cells increases in lupus versus control samples, while additionally finding that the average abundance for non-classical monocytes increases in lupus samples versus controls; see Fig. 5c for a visual summary. Cell types found to be different in average abundance only in the European ancestry were plasmacytoid dendritic cells (pDCs). For the Asian population, we find a decrease of CD4 T-cells (T4) and a decrease in CD8 T-cells (T8). Thus, validation of the original analysis on the CD4 T-cells is only confirmed for Asian ancestry, but not for European ancestry. Interestingly, the original analysis notes that the decrease in CD4 T-cell abundance is higher for Asian cases as compared to European ones, which the authors confirm using external data based on complete blood counts. When testing for an interaction effect between disease and ancestry, we find no significant cell types, but CD4 T-cells are indeed the top ranked cell type, however, with an unadjusted *P*-value of .51 (FDR-adjusted *P*-value of .60).

We note that these results are largely due to a switch in analysis methodology, rather than accounting for the processing cohorts in the statistical analysis. When not accounting for the processing cohorts, in terms of statistical significance on a 10% FDR level, the qualitative results mostly remain for the disease effect within each ancestry, and for the interaction effect, we now do pick up the CD4 T-cells (Fig. 16, available as supplementary data at *Bioinformatics* online). These findings indicate that the CD4 T-cell interaction effect picked up in the original manuscript may partially be driven by technical effects from the processing cohorts.

We contrast our results against two alternative methods, the negative binomial GLM (NB-GLM) and LinDA (see Section 2) for all three contrasts. Detailed results are shown in Fig. 17, available as supplementary data at *Bioinformatics* online. In general, cell types that are discovered by voomCLR are confirmed by other methods. However, other methods often uniquely identify additional cell types as well, possibly reflecting the increased FDR of those methods, which may be alleviated using our bootstrapping approach. Across all 33 evaluations (11 cell types × three contrasts), all methods agree on the DA calling at a 5% FDR level for 27 of them, showing major agreement. The NB-GLM has unique DA calls for four evaluations, while voomCLR and LinDA agree for four evaluations, where NB-GLM has a different result (Fig. 18, available as supplementary data at *Bioinformatics* online).

### 3.5 Human breast cell atlas case study

We also evaluate voomCLR alongside other methodologies on a breast cell atlas case study dataset, for which details can be found in the Supplementary Results, available as supplementary data at *Bioinformatics* online.

## 4 Discussion

We developed voomCLR, a statistical method for the differential analysis of cell compositions, and apply it to simulated and real datasets. It is shown that both the CLR transformation and bias correction are required when modeling cell compositions. Accounting for the uncertainty involved in the bias correction generally improves false positive control. Additionally, the method’s performance is superior in low sample size settings, where it benefits from accounting for heteroscedasticity and adopting empirical Bayes shrinkage of the residual variances.

While our results show that accounting for compositionality should occur in the analysis of cell compositions, we also note that non-compositional models originally developed for differential expression analysis, edgeR, DESeq2, and limma-voom, perform well in settings with a high sample size and high number of cell types. The latter is a natural consequence, as compositional effects get diluted with a high number of features, as is the case in gene expression studies where thousands of features are measured and analysed simultaneously.

Albeit the good performance, one needs to be careful when applying the methodology when the number of features (cell types) are limited. The CLR transformation cannot be expected to work well if the geometric mean is calculated on a very low number of data points, e.g. three cell types. Especially if one or two of these cell types are differentially abundant, it will have a large impact on the geometric mean calculation, which will no longer serve as a reliable reference to compare against. Second, the empirical weights calculation to account for heteroscedasticity relies on a reasonably large number of cell types in order to have a stable lowess fit. This challenge, however, can be tackled via analytical calculation of the weights based on the delta method. This seems to help especially with FDR control, as shown in our simulations. Lastly, the bias correction assumes most cell types are not differentially abundant. This assumption will be more easily violated with a low number of cell types, although performances are still reasonable when the assumption is violated (Fig. 9, available as supplementary data at *Bioinformatics* online). Even so, the uncertainty in the estimation of the bias term increases as the number of cell types decreases. This also applies for the estimation of the variance of the mode as obtained via the bootstrap.

We have shown the added value of accounting for the uncertainty in the estimation of the bias term, using either a non-parametric or parametric bootstrap procedure. In our evaluations, the non-parametric bootstrap tends to render conservative results, especially in the case of a low number of cell types. The parametric bootstrap has the additional advantage of also accounting for the covariance, and in general performs better than the non-parametric bootstrap. Ultimately, we provide both options in the software, and the decision should be guided by the desire of how strict one may want to control false positive results. Alternatively, when the statistical analysis is aimed at discovery of patterns to be validated in future experiments, the analyst may choose not to use the bootstrap to increase power, and filter out false positives in downstream validation.

We have applied the method on single-cell RNA-seq studies, but its application domain should not be limited to this data type. We expect voomCLR to be equally useful for DA testing in flow or mass cytometry and microbiome studies.

## Supplementary Material

btaf637_Supplementary_Data
