# Supplementary material for: Assessing differential cell composition in single-cell studies using voomCLR
Source: Bioinformatics. 2025 Nov 23;42(1):btaf637. doi: 10.1093/bioinformatics/btaf637 (PMC12812216; doi:10.1093/bioinformatics/btaf637)
Supplement: btaf637_Supplementary_Data [file btaf637_supplementary_data.pdf]

Assessing differential cell composition in single-cell studies using voomCLR: Supplementary Material

Alemu Takele Assefa[1], Bie Verbist[1], Koen Van den Berge[1*]

[1] Statistics and Decision Sciences, Johnson and Johnson Innovative Medicine, Beerse, Belgium

[*] To whom correspondence should be addressed: kvande14@its.jnj.com

1 Supplementary Results

1.1 Performance of voomCLR in different configurations

The simulation results in Supplementary Figure S7 compares the performance of voomCLR configurations for an increasing number of cell types. The results indicate that voomCLR with empirical observational weights tends to lose control of FDR when the number of cell types P is less than 10, i.e., when there is a high degree of uncertainty in the empirical mean-variance trend estimation. However, the negative binomial-based analytical weights effectively control the FDR while maintaining a high TPR. For medium to high numbers of cell types, the choice between empirical and negative binomial-based analytical weights does not have a significant impact. On the other hand, Poisson-based analytical weights generally underperformed concerning TPR level in most of the simulation settings.

Generally, the non-parametric bootstrap procedure is conservative in terms of FDR control and therefore comes with an associated cost of reduced sensitivity. On the other hand, the parametric bootstrap is moderately liberal and is associated with higher sensitivity. The simulation study indicated that given voomCLR is applied with a parametric bootstrap procedure (for $P \leq 10$), it is best to use the analytically calculated observational weights based on the negative binomial distribution for optimal performance. In addition, these results indicate that the choice between parametric and non-parametric bootstrap is more important than the choice between Poisson and negative binomial distribution for observation weights. For $P > 10$, the choice between empirical or negative binomial based analytical weights has minimal impact on the performance of voomCLR. As a result, the default most powerful settings are voomCLR either with parametric bootstrap and empirical observational weights (voomCLR (empirical weights & parametric bootstrap)) or parametric bootstrap procedure with analytical observational weights based on the negative binomial distribution (voomCLR (NB analytical weights & parametric bootstrap)) for differential abundance analysis. These conclusions generally apply to the results from both simulation procedures (non-parametric and parametric). However, in the parametric simulation study, where the variability between and within cell populations is higher (Supplementary Figures S2 -S5), the TPR of all methods is relatively lower.

In order to also evaluate the impact of adopting bias correction and empirical Bayes shrinkage of the residual variances, we also evaluated the performance of voomCLR with and without these features. The results shown in Supplementary Figure S8 indicate the heteroscedasticity correction improved the TPR level of voomCLR while the empirical Bayes procedures improved the FDR control. These procedures improve the FDR control of voomCLR only when applied together with the bias correction step. Nevertheless, for a relatively large sample size ($n \geq 20$), none of these procedures contribute significantly to the performance of FDR control and TPR except for the bias correction step.

In conclusion, voomCLR (NB analytical weights & parametric bootstrap) is a good default configuration of the method to be adopted in general, but other configurations can be beneficial in specific circumstances.

1.2 Performance of voomCLR compared to other methods

The results presented in Figure 4 compare the 10 methods discussed above based on a simulation setting with 11 cell populations ($P = 11$) at 3 different simple sizes ($n = 5, 10$ and 20 in each group), similar to Figure 3. Notably, two methods—voomCLR and LinDA—stand out with respect to FDR control compared to all the other methods. Recall that these methods model CLR-transformed data and incorporate bias correction for effect sizes. In contrast, the linear model applied to CLR-transformed counts without bias correction, heteroscedasticity correction, or empirical Bayes shrinkage performs poorly in terms of both FDR control and TPR. Although CLR transformation is widely used to process compositional data, our results suggest that relying solely on CLR transformation for compositional cell count data does not guarantee effective control of false positives when one tests for differential abundance.

In particular, the results in Figure 4 indicate that voomCLR generally outperforms LinDA especially when the sample size is low ($n \leq 10$). This is particularly apparent when the variability between samples is medium to high, and holds even for 10 to 20 samples per group. (Supplementary Figure S10). In simulation scenarios with medium and large variability between samples, voomCLR demonstrates a significant performance advantage in controlling the FDR compared to LinDA even for 10 and 20 samples per group. voomCLR achieves this while maintaining a comparable TPR for detecting truly differentially abundant cell populations. Although both tools leverage the bias correction procedure, voomCLR's superior performance can be explained by its additional incorporation of heteroscedasticity, empirical Bayes shrinkage of the residual variances and accounting for the uncertainty in the bias correction. A mathematical assumption for the bias correction procedure is that the fraction of truly differentially abundant cell populations is not large. However, an additional simulation study spanning low to high proportions of truly differentially abundant cell populations (Supplementary Figure S9) demonstrates that the performance of voomCLR in controlling the FDR remains reasonably well even when the fraction of truly differentially abundant cell populations is large.

Another popular and naturally intuitive approach for testing differential abundance is the negative binomial GLM framework, using the total cell counts as an offset (NB_GLM (TMB)). However, our simulation study shows that this approach generally leads to poor performance, with the actual FDR often exceeding 50% for a nominal 5% FDR (Figure 4 and Supplementary Figure S10). Although it achieved the highest TPR compared to all the methods evaluated in the study, the inadequate FDR control indicates that it is an ineffective procedure for handling compositional data. Methods developed for differential gene expression analysis, edgeR, DESeq2 and limma-voom demonstrated improved performance compared to that of a vanilla NB-GLM. The improved performance of edgeR, DESeq2 and limma-voom can be explained by the normalization procedure these three tools apply on the total cell counts that helps in dealing with compositional data and the empirical Bayes procedure for moderated testing (Supplementary Figure S11). Nevertheless, for a typical number of cell populations that is between 10 and 30, edgeR, DESeq2 and limma-voom underperform compared to voomCLR in terms of FDR control whereas, in simulated data with more than 50 cell populations, these methods perform relatively well and comparable to voomCLR (Supplementary Figure S12). Indeed, these methods performed more on par since the compositional effect decreases when the number of cell populations increases.

Moreover, the simulation study indicates that propeller–a recently introduced tool designed specifically for testing differential abundance in compositional cell population data from single-cell RNA-seq data – generally did not perform well in both simulation settings (Figure 4 and Supplementary Figures S10 and S12). Specifically, the FDR level far exceeds the nominal level indicating its inadequacy for properly handling compositional effects. The choice of transformation techniques (arcsine or logit) did not significantly alter propeller's performance. Similarly, DCATS – also developed for testing differential abundance in compositional cell population data from single-cell RNA-seq data using a beta-binomial GLM framework– ranked equally to propeller with respect to FDR control and TPR in all simulation settings (Figure 4 and Supplementary Figures S10 and S12).

Finally, we repeated the non-parametric simulation study using the healthy samples from the Breast Cell Atlas data as a baseline. The results shown in Supplementary Figure S13 further confirm the superior performance of voomCLR over the other methods evaluated in the study with respect to FDR control. In this particular simulation, all methods generally have lower TPR. However, from assessing the mean-variance trend, it can be seen that there is a larger biological variability in the Breast Cell Atlas data than in the Lupus data (Supplementary Figure S14) which negatively impacts the performance of methods especially at low sample sizes. This result is consistent with the observation from the parametric simulation study with

high simulated biological variability (Supplementary Figure S10). The results in Supplementary Figure S13 also indicate that LinDA performs relatively poorly with respect to FDR control in this simulation study especially when the sample size is 5 per group. Propeller (with both transformation methods) on the other hand showed relatively better performance for FDR control.

1.3 Human breast cell atlas case study

A cell atlas of the human breast was recently published by Reed et al. [2024], where a cohort of healthy breast tissue samples from 55 donors was assessed using single-cell RNA-sequencing. The cohort constitutes 22 women having undergone reduction mammoplasty, 27 samples from women who carry a *BRCA1* or *BRCA2* mutation or had a family history of breast cancer that was not attributed to known risk genes, and 6 *BRCA1*-carrier women that had breast cancer in one breast and had the second breast removed to reduce risk of further tumors [Reed et al., 2024]. A total of $800,000$ single cells were measured, which could be split in three major compartments: stromal, epithelial and immune cells.
Like the original manuscript, we investigated the association of genetic and environmental factors with cell type composition in the breast. In particular, we assessed the impact of genetic risk factors, comparing high-risk *BRCA1*- and *BRCA2*-carriers with average risk donors; see Methods. These contrasts were assessed for the stromal, epithelial and immune cells separately, like in the original manuscript [Reed et al., 2024]. However, here we will focus on immune cells (Figure S1a), as a subset of these results were validated in follow-up experiments, providing a near ground truth. Indeed, enrichment of CD8- and CD4 T-cell types in high risk (HR) donors as compared to average risk (AR) donors was confirmed via immunofluorescence staining. Alongside voomCLR, we also compare with methods LinDA and edgeR as well as the neighborhood-based milo approach [Dann et al., 2022] used in the original paper.

In this comparison, voomCLR and LinDA confirm that CD8 and CD4 T-cell types are increased in both high risk (HR) donor groups as compared to the average risk (AR) donors, while edgeR does not find any significant effects for these cell types (Figure S1b and Supplementary Figure S19); underscoring the importance of accounting for compositionality. For both comparisons, the bias correction term is fairly uncertain, rendering some cell types insignificant at 5% FDR level when using the parametric bootstrap in a voomCLR analysis (Supplementary Figures S20, S21). We also perform a neighborhood-based milo analysis on the immune cells, where we mainly find evidence for increased abundance of the CD8 cell types in the *BRCA1*-carriers as compared to average risk donors (Figure S1c). However, we do not find evidence for the CD4 cell types nor do any cell types have substantial evidence when comparing *BRCA2*-carriers to average risk donors. Several of the milo neighborhoods could not be linked to a single cell type, and are assigned the 'Mixed' label, which may be a possible explanation. We were also unable to construct shared neighborhoods across both comparisons.

When associating immune cell type composition with age and parous status, as in the original manuscript, all methods (voomCLR, LinDA, edgeR and milo) find no significant effects at a 5% FDR level.

2 Supplementary Methods

2.1 Variance of centered log-ratio transformed counts

As before, let Y_{ip} denote the population counts for cell population $p \in \{1, \dots, P\}$ in sample $i \in \{1, \dots, n\}$. If we assume that $\mathbf{Y}_i | N_i, \boldsymbol{\pi}_i \sim Mult(N_i, \boldsymbol{\pi}_i)$, with $\mathbf{Y}_i$ the P-dimensional vector of population counts, $N_i = \sum_{p=1}^{P} Y_{ip}$ the total number of cells for sample i and $\boldsymbol{\pi}_i$ the P-dimensional vector of expected relative abundances. Then, this can be approximated by assuming independent Poisson distributions for each population, i.e., $Y_{ip}|\lambda_{ip} \sim Poi(\lambda_{ip})$, where $\lambda_{ip} = \pi_{ip} \sum_p \lambda_{ip}$ and $\sum_{p=1}^{P} \lambda_{ip} = N_i$. First, note that we can

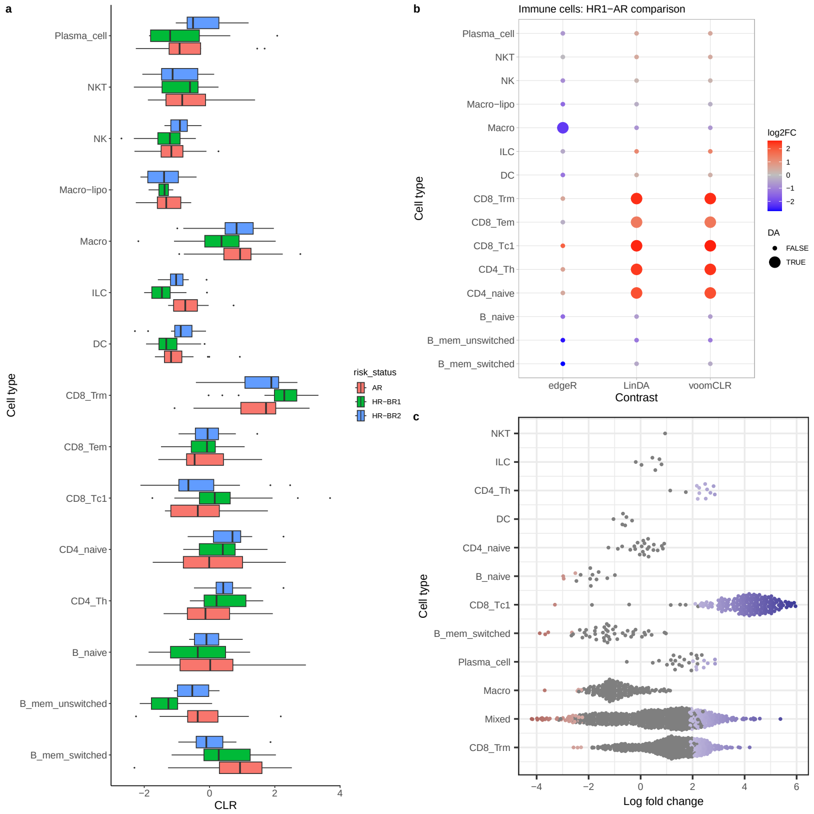

Supplementary Results Figure S1: *Breast Atlas case study.* **(a)** Boxplots of CLR-transformed cell type abundances, for average risk (AR) donors and high risk donors with BRCA1 (HR-BR1) and BRCA2 (HR-BR2) mutations. **(b)** Heatmap showing results of edgeR, LinDA and voomCLR without bootstrapping, for all cell types (y-axis) in the comparison of HR-BR1 versus AR donors. Each point is colored according to the log2 fold-change (positive is higher abundance in HR-BR1 donors), and point size denotes whether the FDR-adjusted p-value is below the 5% level. **(c)** Milo results for all cell types, where each data point represents a neighborhood. Neighborhoods are assigned to a cell type as soon as at least 70% of its constituent cells are from that cell type. Significant neighborhoods (5% FDR level) are colored, while insignificant ones are in grey.

rewrite the CLR transformation as

$$\begin{aligned} Z_{ip} &= \log \frac{Y_{ip}}{\exp\left\{\frac{1}{P}\sum_{p=1}^{P}\log Y_{ip}\right\}} \\ &= \log\left[Y_{ip}\exp\left(-\frac{1}{P}\log Y_{ip}\right)\exp\left(-\frac{1}{P}\sum_{q\neq p}\log Y_{iq}\right)\right] \\ &= \frac{P-1}{P}\log Y_{ip} - \frac{1}{P}\sum_{q\neq p}\log Y_{iq}. \end{aligned}$$

The variance of CLR-transformed counts can be approximated using the Delta method [Dorfman, 1938, Doob, 1935] based on the Poisson assumption.

$$
\begin{aligned}
&\text{If } Y_{ip} \sim Poi(\lambda_{ip}) : \\
Var[f(Y_{ip})] = Var(Z_{ip}) &\approx Var(Y_{ip})\left\{f'(E[Y_{ip}])^2\right\} \\
&= \lambda_{ip}\left(\frac{P-1}{P\lambda_{ip}}\right)^2 \\
&= \left(\frac{P-1}{P}\right)^2 \frac{1}{\lambda_{ip}}.
\end{aligned} \tag{1}
$$

The Multinomial assumption may be too restrictive, therefore several methods rely on a Dirichlet-Multinomial distribution to model cell counts. In turn, the Dirichlet-Multinomial distribution across cell populations can be approximated by assuming a negative binomial distribution for each cell population, i.e., $Y_{ip} \sim NB(\mu_{ip}, \phi_p)$. Based on this, the Delta method would then approximate the variance as

$$
\text{If } Y_{ip} \sim NB(\mu_{ip}, \phi_p) : Var(Z_{ip}) \approx \left(\frac{P-1}{P}\right)^2 \left(\frac{1}{\mu_{ip}} + \phi_p\right). \tag{2}
$$

2.2 Parametric bootstrap procedure

A linear model on CLR-transformed cell type counts is fitted,

$$
Z_{ip} = \beta_{0p} + \sum_{j=1}^{k} \beta_{jp} C_{ij} + e_{ip}, \tag{3}
$$

and we are interested in statistical inference on (a linear combination of) the mean parameter(s) β_{jp}. Analogous to the non-parametric bootstrap procedure, we develop a parametric bootstrap to accommodate the uncertainty involved in estimation of the bias correction term. For each population p, we sample the $(k+1) \times 1$ vector $\hat{\boldsymbol{\beta}}_p$ (by default 4000 times) from a multivariate normal (MVN) distribution

$$
\hat{\boldsymbol{\beta}}_p^B \sim MVN(\hat{\boldsymbol{\beta}}_p, \hat{\Sigma}_{\hat{\beta}_p}), \tag{4}
$$

where $\hat{\Sigma}_{\hat{\beta}_p} = \tilde{\sigma}^2(\mathbf{C}^T\mathbf{W}\mathbf{C})^{-1}$, with $\tilde{\sigma}^2$ the moderated residual variance estimate [Smyth, 2004, Law et al., 2014], $\mathbf{C}$ the $n \times (k+1)$ design matrix from the model in Equation (3) and $\mathbf{W}$ a $n \times n$ diagonal weight matrix corresponding to the `voom` heteroscedasticity weights [Law et al., 2014]. Let $\beta_{cp} = \mathbf{L}_c\boldsymbol{\beta}_p$ denote a contrast c of interest, where $\mathbf{L}_c$ is a $1 \times (k+1)$ contrast vector and $\boldsymbol{\beta}_p$ the $(k+1) \times 1$ vector of regression coefficients. Note that calculating the contrast for a given bootstrap sample, $\hat{\beta}_{cp}^B = \mathbf{L}_c\hat{\boldsymbol{\beta}}_p^B$, is equivalent to sampling from a univariate Normal distribution of the form

$$
\hat{\beta}_{cp}^B \sim N(\mathbf{L}_c\hat{\boldsymbol{\beta}}_p, \mathbf{L}_c\hat{\Sigma}_{\hat{\beta}_p}\mathbf{L}_c^T). \tag{5}
$$

For each contrast of interest, we then calculate the corresponding mode in each bootstrap sample, i.e., $\check{\beta}_c^B$. Estimating the variance across bootstrap samples and statistical inference then proceeds as in the non-parametric bootstrap procedure.

2.3 Simulation frameworks

2.3.1 Parametric simulation using a Dirichlet-Multinomial distribution

The Multinomial distribution is a natural choice for modeling compositional cell population counts in various contexts. Nevertheless, it falls short in fully explaining the inherent variability observed between biological replicates, a phenomenon known as overdispersion. To address this issue, it is common to treat the Multinomial probabilities as random variables drawn from a Dirichlet distribution to effectively capture the overdispersion. In this study, compositional count samples are generated using a hierarchical Dirichlet-Multinomial distribution. This allows for a flexible and realistic simulation of compositional cell population

data, accommodating the inherent variability in biological replicates more accurately. Below, we outline the procedure used for the simulation.

The objective is to simulate compositional cell count data for P populations (cell types) measured on two independent groups of samples with replicate size n_1 and n_2. Let $\mathbf{Y}_i$ be a P-dimensional vector, representing the count vector of a sample $i \in \{1, \ldots, n\}$. We assume $\mathbf{Y}_i \sim \mathcal{DM}(\boldsymbol{\pi}_i, \boldsymbol{\theta}_i, N_i)$, where $\boldsymbol{\pi}_i$ and $\boldsymbol{\theta}_i$ are P-dimensional vectors of Multinomial probabilities of sample i and Dirichlet parameters, respectively. For population p, we define the Dirichlet parameter $\theta_{ip} = \gamma \exp\{\beta_{0p} + X_i\beta_{1p}\}$, where $X_i = 1$ if sample i is from group 2 and 0 if from group 1. Note that the Dirichlet parameters are the same for all samples from the same group. We denote v_0 and v_1 to be two sets of cell types ($|v_0| + |v_1| = P$ and $v_0 \cap v_1 = \emptyset$) for which the null and alternative hypothesis holds, respectively. Note that for a population $p \in v_0$, its expected absolute count is the same between the two groups of samples. The parameter β_{1p} controls the magnitude of differential abundance between the two groups of samples for population $p \in v_1$ and the multiplication factor $\gamma > 0$ controls the level of variability among samples, which is set constant for both sample groups. A low value of γ results in large variability between samples. The vectors $\boldsymbol{\beta_0}$ and $\boldsymbol{\beta_1}$ are configured to exhibit variation across cell populations by being sampled from normal distributions. Specifically, $\beta_{0p} \sim N(1, \tau_0)$, where τ_0 governs the variability between cell populations in terms of abundance levels. In our simulation study, we chose $\tau_0 = 2$ to generate cell population data that mirrors the variability observed in the Lupus case study dataset. Similarly, for β_{1p}, we employed $\{p \in v_1 : \beta_{1p} \sim N(0, \tau_1)\}$, where $\tau_1 = 2$ to simulate effect sizes.

Given $\boldsymbol{\theta}_i$, the Multinomial probabilities for sample i are simulated from a Dirichlet distribution. That is, $\boldsymbol{\pi}_i|\boldsymbol{\theta}_i \sim \mathcal{D}(\boldsymbol{\theta}_i)$. Subsequently, for sample i, using the obtained $\boldsymbol{\pi}_i$ and the desired total number of cells N_i, the count vector $\mathbf{Y}_i$ is sampled from a Multinomial distribution. That is, $\mathbf{Y}_i|\boldsymbol{\pi}_i, N_i \sim \mathcal{M}(N_i, \boldsymbol{\pi}_i)$. The simulated data can be denoted by an $n \times P$ matrix $\mathbf{Y}$. Data were generated under the following scenarios:

1. Low ($\gamma = 1.5$), medium ($\gamma = 1$) or high ($\gamma = 0.25$) levels of variability between samples by controlling the γ parameter.
2. Different sample sizes $n_1 = n_2 \in \{5, 10, 20\}$.
3. Different number of cell types $P \in \{5, 10, 20, 50, 100\}$.
4. Different number of truly differentially abundant cell types (pDA): 10, 20, 50, 75 and 85% of the number of cell types (P)

In most of the simulation settings, the number of truly differentially abundant populations $|v_1|$ is set to be 20% of P (rounded to the nearest higher integer number) unless specified otherwise.

2.3.2 Non-parametric simulation framework

Although simulation from Dirichlet-Multinomial distribution provides flexibility to simulate a wide range of scenarios, the parametric assumptions inherent in modeling cell count data may not always align with reality. In recognition of this limitation, and to steer clear of such parametric assumptions, we adopt a novel distribution-free approach. This alternative method begins with real cell population data, enabling the simulation of realistic new datasets with a built-in truth. Below, we outline the procedure.

This simulation method uses real data as input. We start with a set of samples between which we expect no systematic biological signal, for example, a group of healthy patients from the lupus cell population dataset. In brief, the simulation method works by first randomly splitting the samples (patients) into two equally sized groups. Again, we expect no systematic signal between these groups. The introduction of signal happens by replacing the cell counts of a randomly selected population with the cell counts of another randomly selected population, within each sample for one of the two artificially created groups. In order to simulate the compositional effect without changing the total number of cells for each sample, we make a compositional correction for all other populations that were not changed.

More formally, let $\mathbf{Y}$ be the $n \times P$ cell population count matrix that serves as the basis for the simulation, with elements Y_{ip}, $i \in \{1, \ldots, n\}$, $p \in \{1, \ldots, P\}$. We randomly split the n samples into two mutually exclusive groups of size n_1 and n_2, where $n_1 + n_2 \leq n$. Let $\mathcal{G}_1$ and $\mathcal{G}_2$ denote the set of samples belonging to group 1 and group 2, respectively. Let $\mathcal{V} = \{(p, q) : p, q \in \{1, \ldots P\},$ and $p \neq q\}$ be a set of indices for a pair of cell populations.

1. Randomly select $\mathcal{V}$. Let $\mathbf{y}_p$ and $\mathbf{y}_q$ denote the n_2-dimensional vectors of cell counts for all samples in $\mathcal{G}_2$ for populations p and q, respectively. Additional notes on the selection of $\mathcal{V}$ are given below.

2. Cell count replacement/swap. From the set $\mathcal{V}$, population p was selected to have signal introduced, and the counts are replaced with those of population q in the second group, i.e., $\mathbf{y'}_p = \mathbf{y}_q$, where $\mathbf{y'}_p$ is the new cell count vector of population p.

3. Calculate spillover cell count: Let $\mathbf{d}_p = \mathbf{y'}_p - \mathbf{y}_p$ is an n_2-dimensional vector denoting the difference in the cell counts of population p introduced by the count replacement in Step 2.

4. Calculate compositional effect: For all other populations $k \neq p$, we calculate the compositional effect caused by replacing population p by population q as $\mathbf{c}_{k(p)} = -\mathbf{w}_{k(p)} \odot \mathbf{d}_p$, where $\mathbf{w}_{k(p)}$ is the wight vector with element $w_{k(p)i}$ for sample $i \in \mathcal{G}_2$ defined as $w_{k(p)i} = \frac{\hat{\pi}_{ki}}{\sum_{k \neq p} \hat{\pi}_{ki}}$, $\hat{\pi}_{ki}$ is the estimated fraction of cell population k in sample i, and $\odot$ denotes element-wise multiplication. See the notes below for additional details.

5. Correct for compositional effect. Add the compositional effect for every cell population $k \neq p$, i.e., $\mathbf{y}'_k = \mathbf{y}_k + \mathbf{c}_{k(p)}$.

6. Repeat steps 1-5 until a desired number of cell populations is set to be differentially abundant.

Note 1: In Step 1, two populations can be selected randomly but to simulate realistic data, we select pairs of populations with probability sampling. The sampling probability for a pair of cell populations p and q is inversely proportional to the Euclidean distance between $\mathbf{x}_p$ and $\mathbf{x}_q$, where $\mathbf{x}$ is the CLR transformed vector.

Note 2: In Step 4, the weights are introduced to redistribute excess counts to other cell populations proportional to their relative abundance. This, for example, keeps a rare population being rare in the simulated data.

One may consider swapping, for instance, two cell populations within the second group of samples to introduce a signal. However, the objective is to simulate realistic cell population data in a manner where the introduction of differential abundance signals in a specific cell population systematically influences the abundance of other cell populations. This process should ultimately emulate the natural data-generating mechanism of cell population data.

The non-parametric simulation is conducted using one of the two real datasets as baselines: (1) all biological replicates from the healthy patients in the lupus dataset from processing cohort 4, or (2) all biological replicates from the healthy groups in the Breast Cell Atlas data. Unless specified, a differential abundance signal is introduced to 20% of the cell populations in each simulated dataset.

2.3.3 Performance assessment metrics

The simulated data contain built-in truth about the truly differentially abundant cell populations. This helps to quantify the actual false discovery rate (FDR) and true positive rate (TPR) for a given nominal level of FDR. The actual FDR is defined as the average false positive fraction among the positives (FP/(FP+TP)) across the simulation runs. The TPR is defined as the average true positive proportions (TP/TP+FN)), where FN, FP, and TP denote, respectively, the numbers of false negatives, false positives, and true positives. FDR-TPR curvesare calculated using the iCOBRA Bioconductor R package [Soneson and Robinson, 2016].

2.4 Lupus scRNA-seq data case study

The lupus scRNA-seq dataset [Perez et al., 2022] was downloaded from the Gene Expression Omnibus using accession number GSE174188. We use the annotation from the paper to create the cell population count matrix, consisting of 11 cell populations and 355 samples. We remove four samples from African American patients and 3 samples from Hispanic patients, focusing the analysis on the remaining samples from Asian (n=137) and European (n=211) ancestry. Out of the remaining 348 samples from 256 individuals, 188 individuals were sequenced once, while 49 individuals were sequenced twice, 14 individuals were sequenced three times, and 5 individuals were sequenced four times. The sequencing happened in multiple phases,

denoted as four different processing cohorts in the original manuscript. We perform a principal components analysis (PCA) on the cell count matrix based on Aitchison's distance (i.e., Euclidean distance on CLR-transformed counts), respecting the compositional nature of the counts. When modeling the counts, we use the same design matrix for all methods, which includes a fixed effect for processing cohort and main and interaction effects for disease status and ancestry. We account for repeated measurements through repeated sequencing by using a random subject effect in the negative binomial model and LinDA. For voomCLR, we approximate the same random effect using the `duplicateCorrelation` functionality of limma [Smyth, 2004]. Statistical inference is focused on the fixed effect part of the model, where we test for disease effect for European ancestry, disease effect for Asian ancestry, and the interaction effect.

2.5 Breast cell atlas case study

The breast atlas case study dataset was downloaded from the cellxgene portal link that was provided in the manuscript [Reed et al., 2024]. Cells belonging to clusters 'Doublet', 'Stripped nuclei', 'DDC1' and 'DDC2' (donor-derived clusters 1 and 2) were removed. The immune cells were obtained by first selecting the 'Stroma enriched' samples and then subsetting to the immune cell types. As in the original manuscript, the associations of age and parity were done on the mammoplasty samples. These tests were always performed with both variables in the model (e.g., the analysis testing for age also included parity as a variable in the model). Samples with no information on parity status were removed. When comparing high-risk with average risk donors, we also condition on age and parity. Analysis was performed separately on the samples consisting of {AR, HR-BR1} and {AR, HR-BR2} data subsets. In the milo analysis of the high risk versus average risk comparison, we were unable to use shared neighborhoods across these comparisons for the downstream statistical inference, since the software errored in the inference evaluation.

2.6 voomCLR software implementation

A software implementation of the proposed methodology has been developed as R package. The code required for running a cell composition analysis is similar to a limma-voom analysis, as our implementation reuses functions and code chunks from the limma software [Smyth, 2004]. In particular, in voomCLR, we adapt the original `voom` function for estimating the mean-variance trend to adopt the CLR transformation and calculating the mean-variance trend for CLR-transformed counts. Model fitting, setting up contrasts and empirical Bayes shrinkage of the residual variance occurs via the native `lmFit`, `contrasts.fit` and `eBayes` functions from limma. For statistical inference, we leverage the code from limma's `topTable` function and create a new function, `topTableBC`, which internally implements the bias correction as implemented in the linDA software and allows propagating bias correction uncertainty via bootstrapping.

The original `voom` implementation estimates the mean-variance trend empirically, using a lowess trend [Law et al., 2014], which is then leveraged to calculate heteroscedasticity weights to be used in linear model fitting. This is not robust with a low number of features (in our case, cell types). The `voomCLR` function allows for an alternative estimation of heteroscedasticity weights by analytically calculating them using the Delta method, using the derivations shown in Methods. The user has the option to choose between variance approximation based on the Poisson or negative binomial approximation.

The software package is publicly available on GitHub at https://github.com/koenvandenberge/voomCLR and will be submitted to Bioconductor.

2.7 Implementation of other methods

2.7.1 Negative Binomial GLM (NB_GLM)

Cell abundance counts are used as a response in a negative binomial generalized linear model (NB GLM), using the `glmmTMB` package (v1.1.9) [Brooks et al., 2017]. An offset corresponding to the logarithm of each sample's total number of cells is used.

2.7.2 edgeR

Similar to NB_GLM, edgeR (v3.38.4) is used to fit negative binomial models to the cell abundance counts of each cell type [Robinson et al., 2010]. The offset now corresponds to the logarithm of normalized total cell counts, with normalization factors calculated based on TMM normalization [Robinson and Oshlack, 2010]. Dispersion parameters are estimated using the `estimateDisp` function and model fitting happens through `glmFit`. In the simulation study, the Quasi-Likelihood (QL) method (`glmQLFit`) is used for testing differential abundance and dispersion estimates obtained using the `estimateQLDisp` function.

2.7.3 DESeq2

We also use DESeq2 (v1.36.0) for negative binomial model fitting, where the total cell counts are now normalized according to the DESeq2 median-of-ratios normalization [Love et al., 2014]. Further, we run DESeq2 using default settings.

2.7.4 limma-voom (limma-voom)

In limma-voom, cell abundance counts are transformed to $\log_2$ counts-per-million, which are then used as response in a weighted linear model. The weights are calculated based on an empirical mean-variance trend as described in [Law et al., 2014], and residual variances are shrunken using empirical Bayes [Smyth, 2004]. The `limma` package (v3.52.3) is used for the implementation of limma-voom

2.7.5 CLR-based linear model (LM_CLR)

In the LM_CLR method, we fit linear models for each cell population, using the CLR-transformed cell counts as response variable. Statistical inference is carried out using t-tests.

2.7.6 LinDA

LinDA is designed for analyzing microbiome compositional data. It fits linear regression models on centered log-ratio transformed data, identifies bias terms, and corrects these biases using the mode of the regression coefficients. The package `LinDA` (v0.2.0) is used with the default settings.

2.7.7 propeller

propeller [Phipson et al., 2022] is a method proposed for testing differential abundance in compositional cell population data. It applies a transformation (using logit or arcsin square root) on cell counts and fits linear models. Empirical Bayes is used for moderated cell type-specific variance estimation. We try both transformations in the simulation study, with all other parameters kept at the default settings. The `fitPropeller` function from the `speckle` (v1.4.0) R package is used.

2.7.8 DCATS

DCATS [Lin et al., 2023] is an R software package developed for differential composition analysis in single-cell RNA sequencing (scRNA-seq) data, with a focus on addressing the uncertainty in cell type assignment. It uses a beta-binomial regression model to analyze raw cell counts, considering dispersion between samples. DCATS corrects for misclassification bias using a similarity matrix between cell types. The package DCATS (v1.2.0) is applied with its default settings.

2.7.9 sccomp

sccomp [Mangiola et al., 2023] is another R software package for differential composition and variability analysis for single-cell data. It uses a Bayesian hierarchical Dirichlet–multinomial model to handle compositional counts. This allows it to capture both the fixed-sum constraint and extra between-sample variability. Inference on the group mean comparison is done by the false-discovery rate calculated using the probability of the null hypothesis (the posterior probability of the fold-change being larger or smaller than the default threshold of 0.2). The false-discovery rate output was used to calculate the performance assessment metrics

in our simulation study. Based on the recommendation in the vignette of the package, the method is applied with a setting$bimodal_mean_variability_association = TRUE$.

3 Supplementary Figures

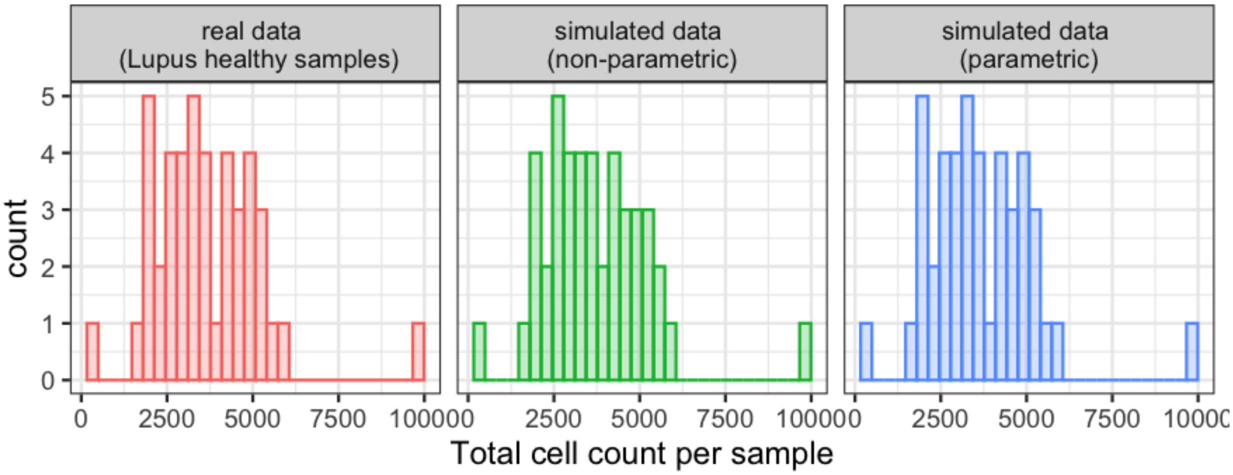

Supplementary Figure S1: *Simulation study:* Comparison of the distribution of the total cell counts per sample between real data (lupus healthy samples from processing cohort 4) and simulated data (**non-parametrically** and **parametrically** simulated data). Note that the distributions from the real and parametrically simulated data are identical because the parametric simulation procedure uses the total cell count from the real data to simulate cell counts from the Dirichlet-Multinomial mixture distribution.

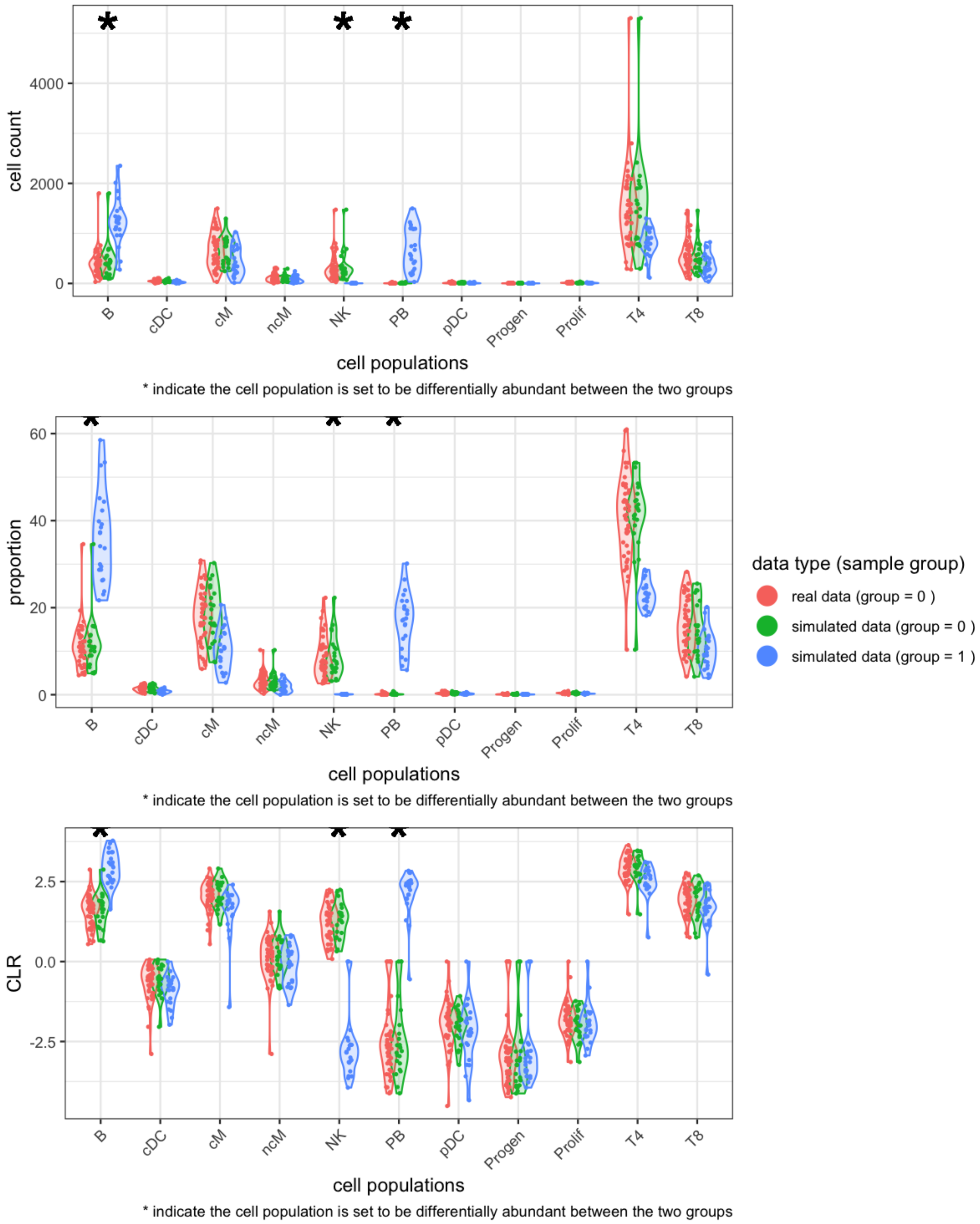

Supplementary Figure S2: *Simulation study:* Comparison of the distribution of the cell counts, proportions, and CLR-transformed counts between real data (lupus healthy samples from processing cohort 4) and **non-parametrically** simulated data.

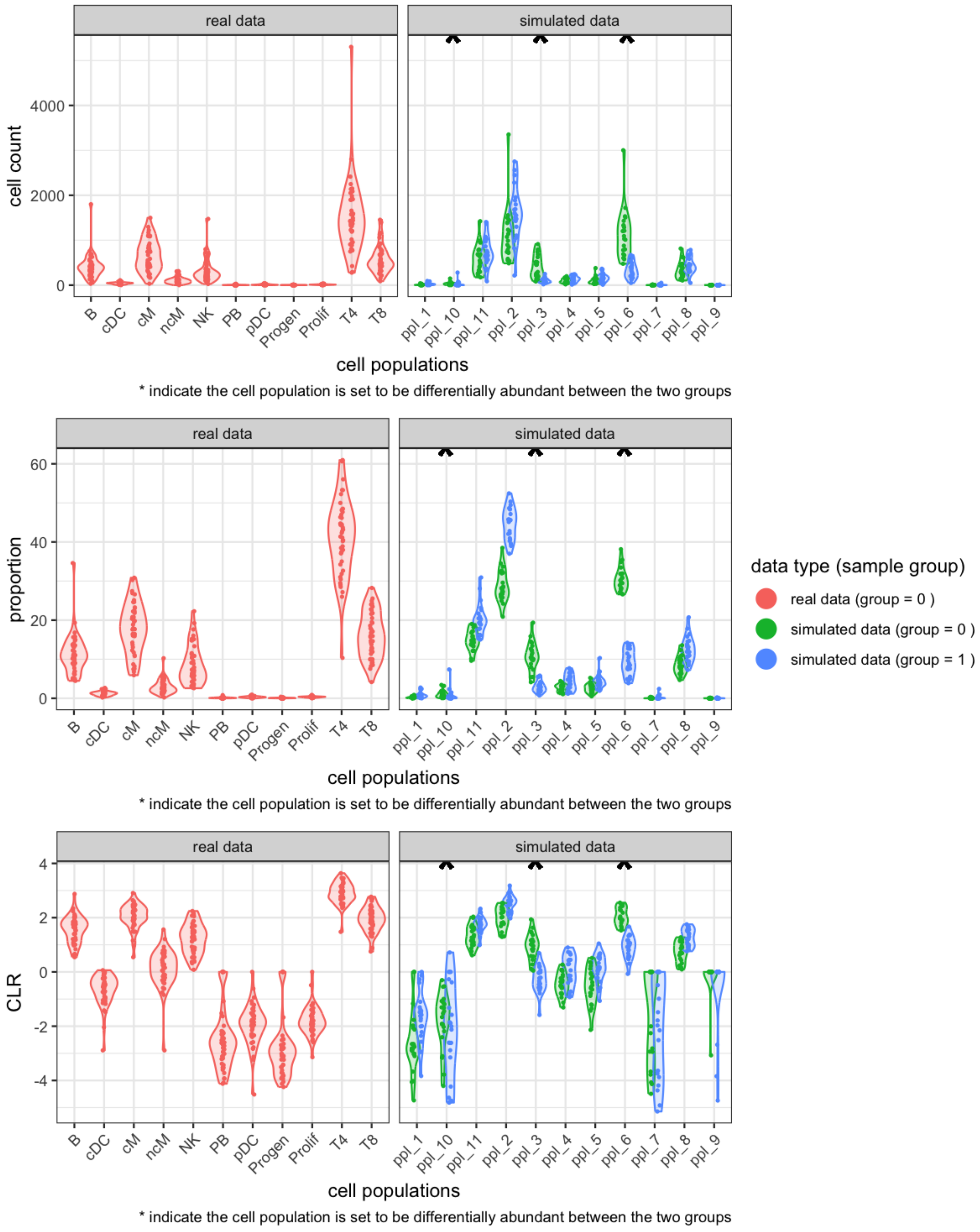

Supplementary Figure S3: *Simulation study:* Comparison of the distribution of the cell counts, proportions, and CLR-transformed counts between real data (lupus healthy samples from processiong cohort 4) and **parametrically** simulated data.

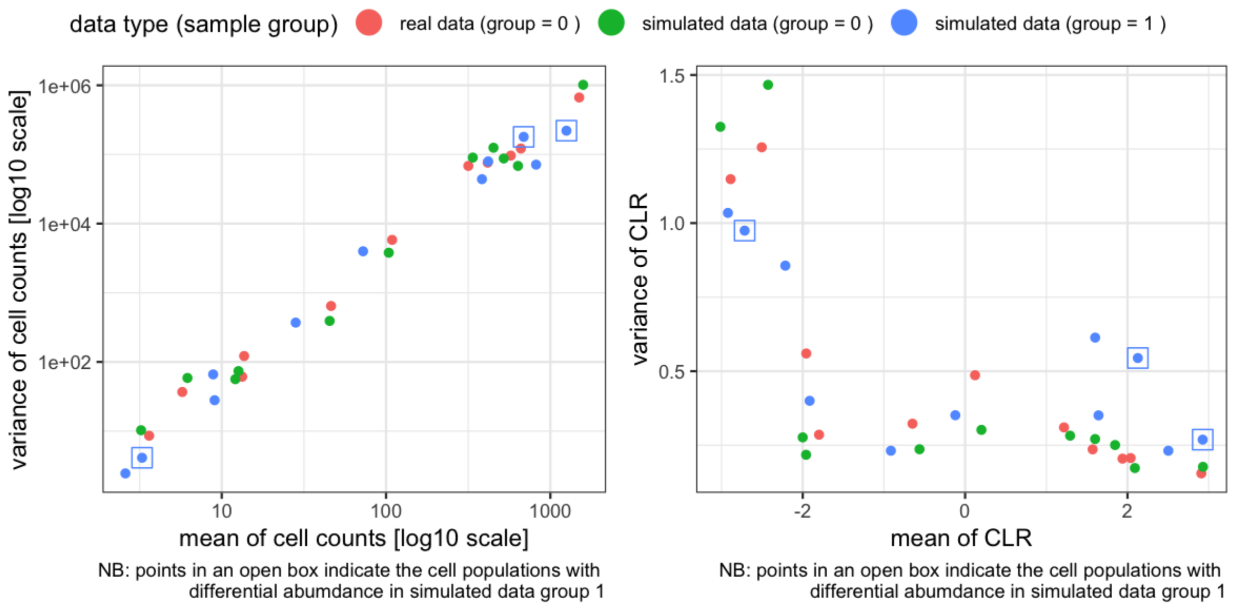

Supplementary Figure S4: *Simulation study:* Comparison of the mean-variance trend for the cell counts and CLR-transformed counts between real data (lupus healthy samples from processing cohort 4) and **nonparametrically** simulated data.

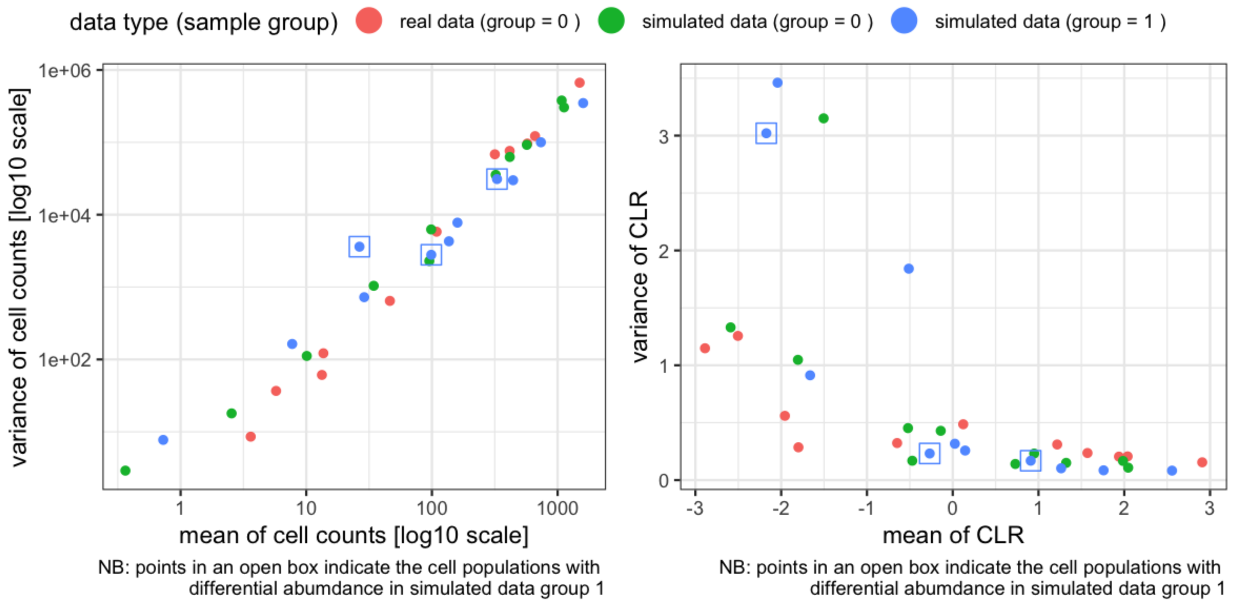

Supplementary Figure S5: *Simulation study:* Comparison of the mean-variance trend for the cell counts and CLR-transformed counts between real data (lupus healthy samples from processing cohort 4) and **parametrically** simulated data.

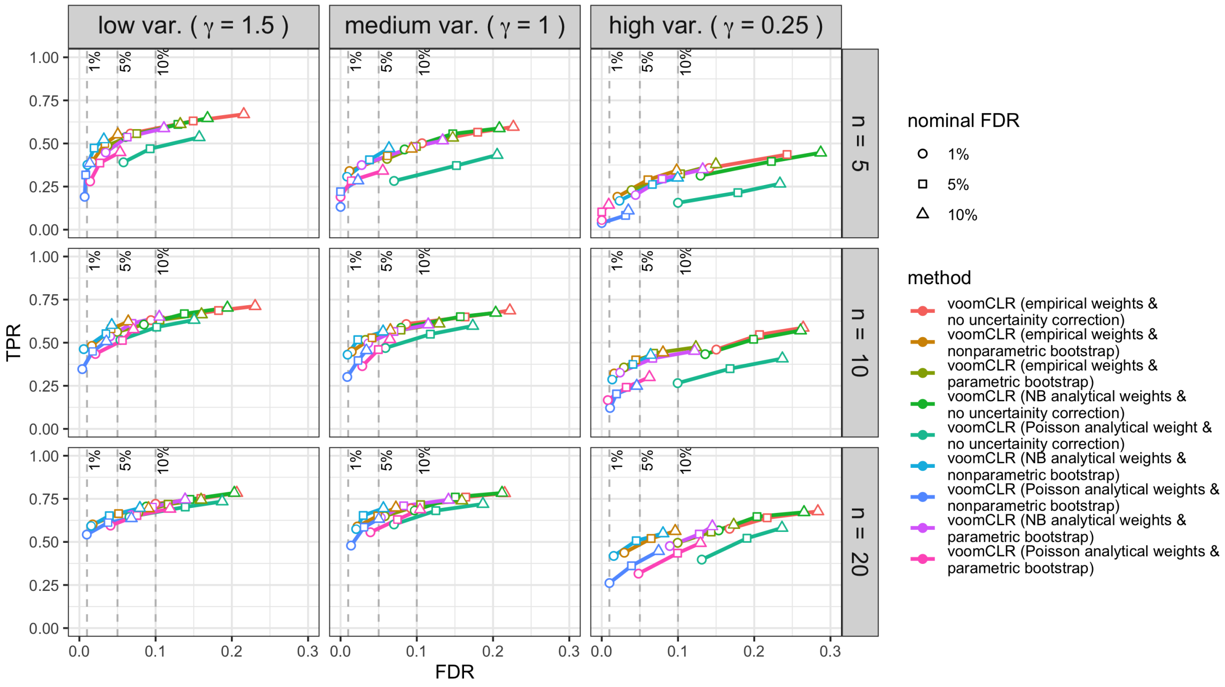

Supplementary Figure S6: *Simulation study:* FDR-TPR curves for evaluating the performance of voomCLR with different configurations in simulated cell population data with three different levels of variability among samples and three different sample sizes. The data is simulated from a Dirichlet-Multinomial distribution, with 3 different levels of variability (low, medium and high) controlled by a scaling factor γ for the Dirichlet parameters and 3 different sample sizes per group ($n = 5, 10$ and 20). Reported metrics are averages from 250 simulation runs for each simulation scenario and performance metrics are calculated at $1, 5,$ and 10% nominal FDR. Each simulated data consists of two independent groups of samples for 11 populations.

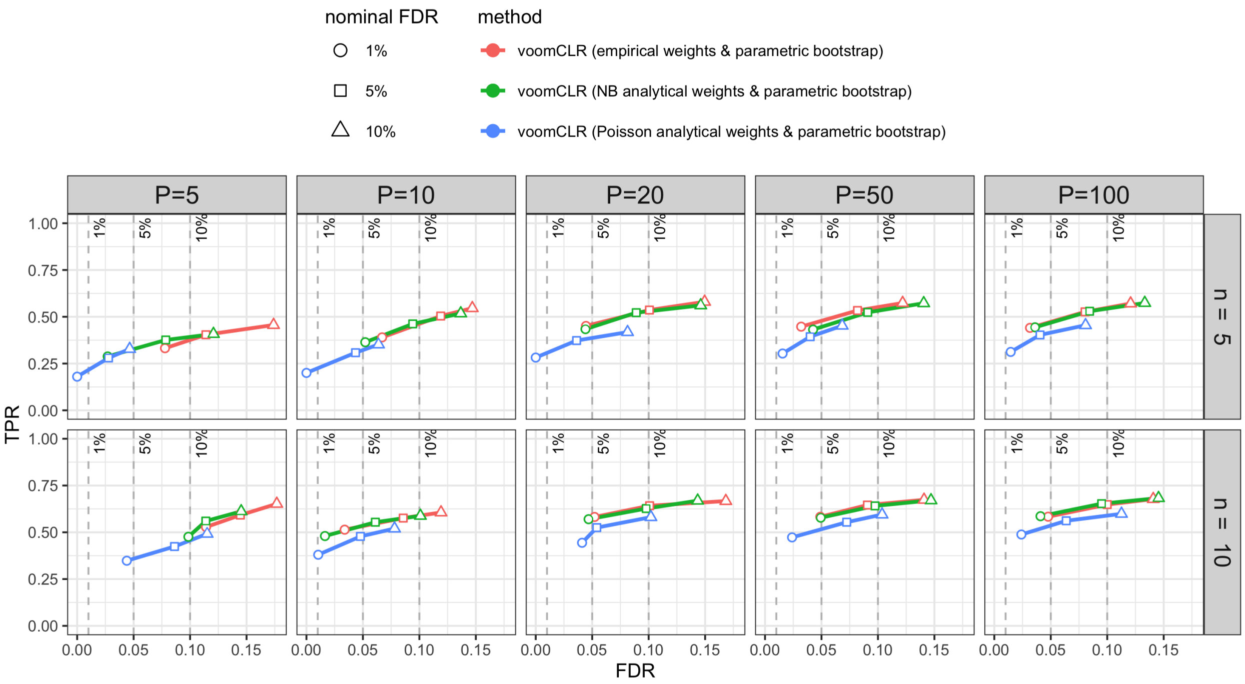

Supplementary Figure S7: *Simulation study:* FDR-TPR curves for evaluating the performance of 3 configurations of voomCLR with respect to estimation of observational heteroscedasticity weights (empirical or analytical weights from negative binomial or Poisson distribution all with parametric bootstrap) in simulated cell population data for 5 different numbers of cell populations ($P = 5, 10, 20, 50$ and 100). The data is simulated from a Dirichlet-Multinomial distribution, with medium level of variability (Dirichlet parameters scaling factor $\gamma = 1$), and 2 different sample sizes per group ($n = 5$ and 10). Reported metrics are averages from 250 simulation runs for each simulation scenario and performance metrics are calculated at 1%, 5%, and 10% nominal FDR.

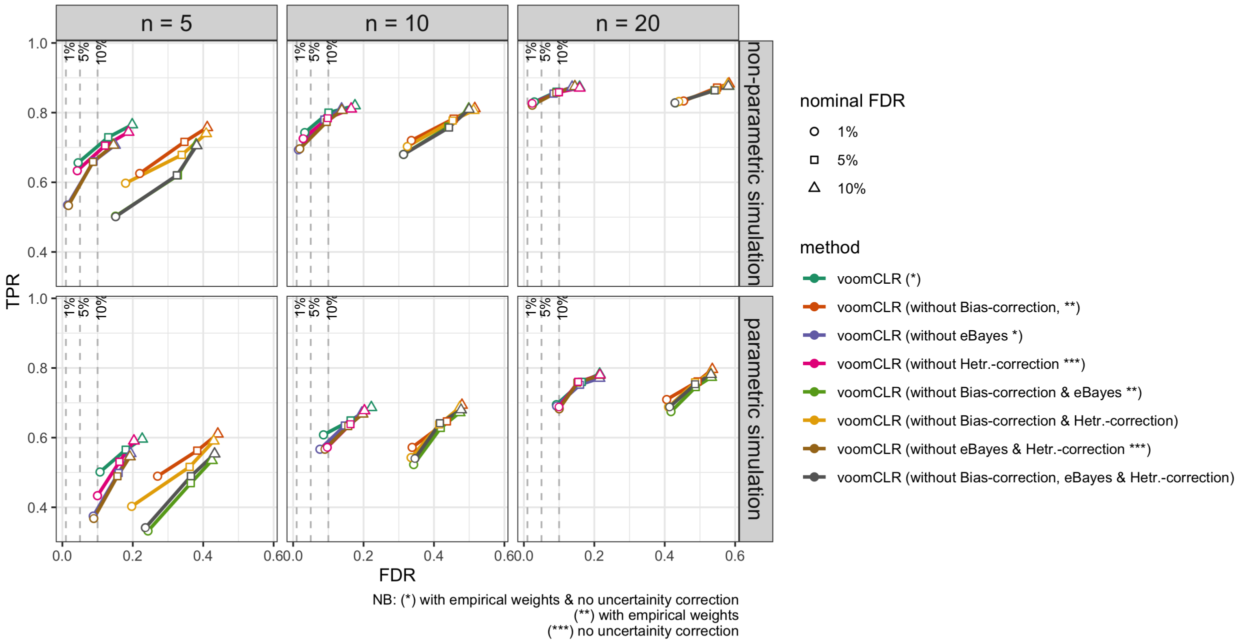

Supplementary Figure S8: *Simulation study:* FDR-TPR curves for evaluating the performance of voomCLR with and without either bias correction, heteroscedasticity or empirical Bayes shrinkage in simulated cell populations with $P = 11$ cell populations. The non-parametric simulation uses the healthy samples from the Lupus dataset in processing cohort 4 as a baseline. The data is simulated using the non-parametric and parametric simulation procedures. Reported metrics are averages from 250 simulation runs for each simulation scenario. The reported performance metrics are at 1%, 5%, and 10% nominal FDR. Notes: annotation in legend "without Bias-correction" is for voomCLR without bias correction, "without eBayes" is for voomCLR without empirical Bayes shrinkage and "without Hetr.-correction" is for voomCLR without accounting for mean-variance trend and all combinations thereoff. Also note that the voomCLR configuration used in these settings is (if applicable) with empirical observational weights and parametric bootstrap procedure.

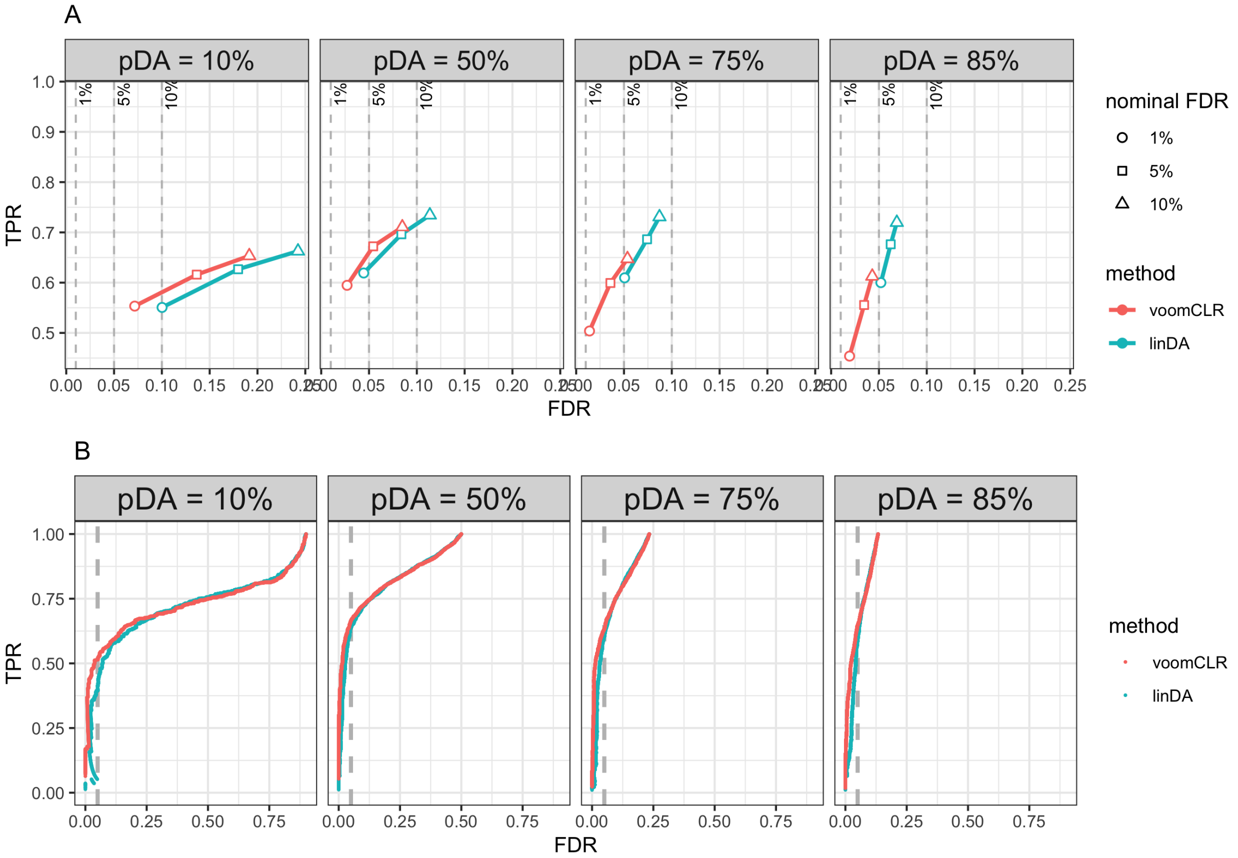

Supplementary Figure S9: *Simulation study:* Performance curves for evaluating the performance of voomCLR and LinDA for in simulation settings with 10, 50, 75 and 85% of the cell populations are set to be DA. The data is simulated from a Dirichlet-Multinomial distribution, with a medium level of variability (Dirichlet parameters scaling factor $\gamma = 1$), and 10 samples per group ($n = 10$). Reported metrics are averages from 250 simulation runs for each scenario. (A) FDR-TPR curves, where the metrics are calculated at 1%, 5%, and 10% nominal FDR, and (B) ROC curves, where the metrics at 5% nominal FDR are shown indicated with the circle.

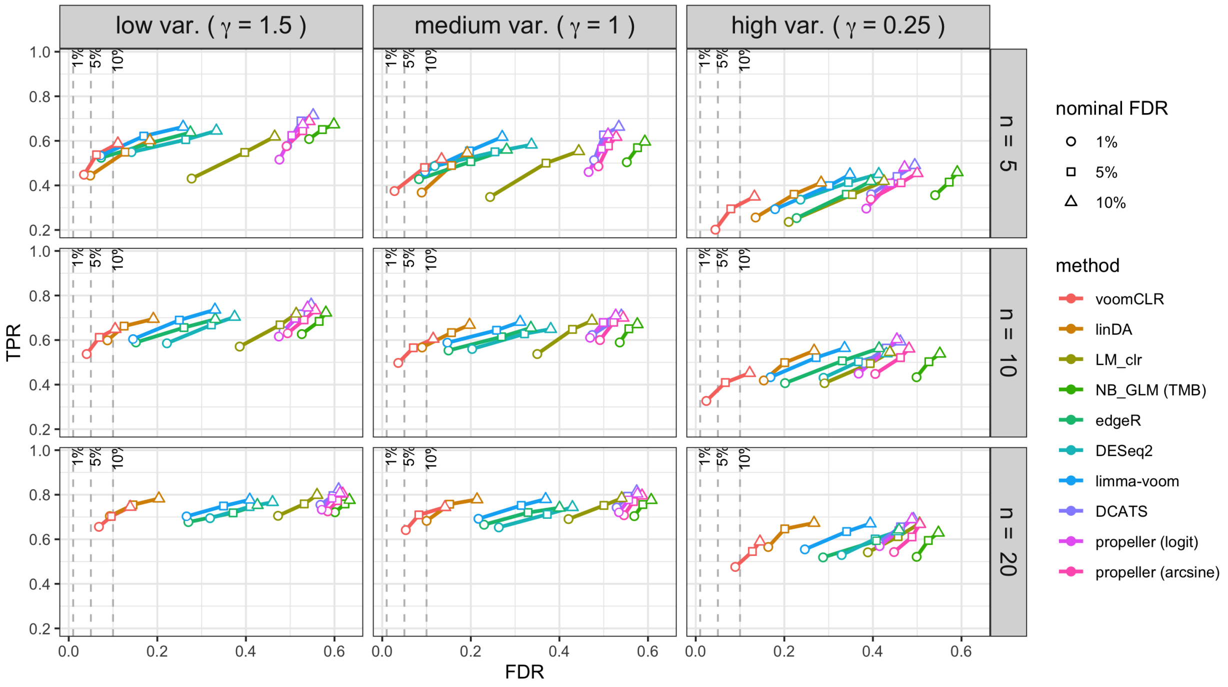

Supplementary Figure S10: *Simulation study:* FDR-TPR curves for evaluating the performance of voomCLR and other methods in simulated cell population data with three different levels of variability among samples and three different sample sizes. The data is simulated from a Dirichlet-Multinomial distribution, with 3 different levels of variability (low, medium and high) controlled by a scaling factor γ for the Dirichlet parameters and 3 different sample sizes per group ($n = 5, 10$ and 20). Reported metrics are averages from 250 simulation runs for each simulation scenario and performance metrics are calculated at $1, 5,$ and 10% nominal FDR. Each simulated dataset consists of two independent groups of samples for 11 populations.

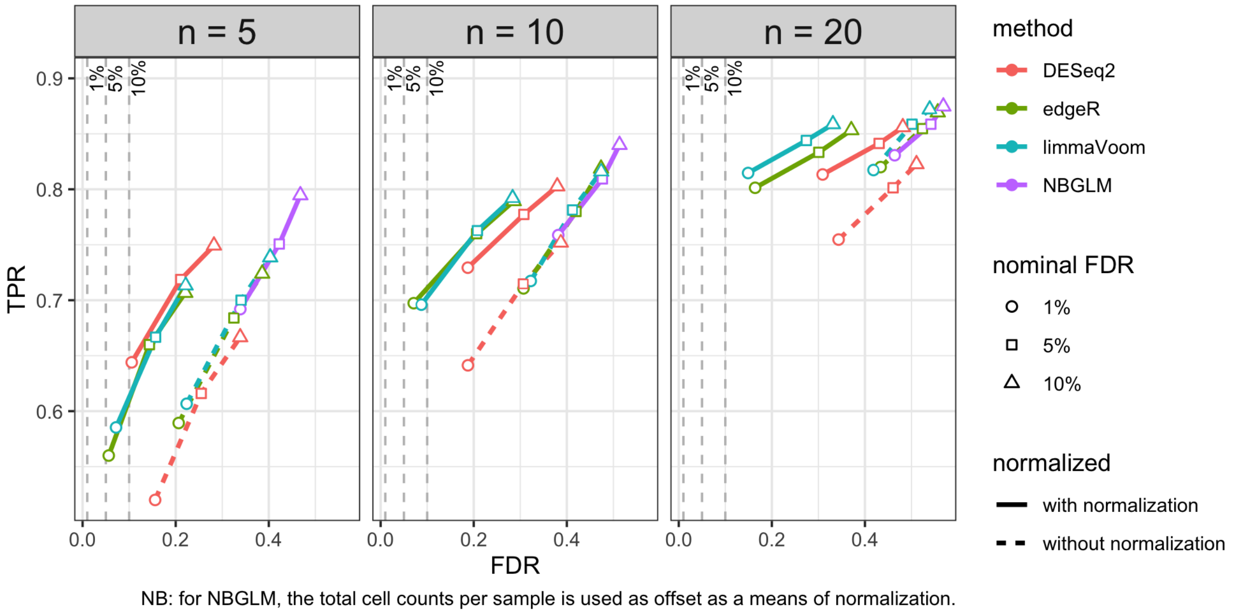

Supplementary Figure S11: *Simulation study:* FDR-TPR curves for evaluating the effect of library size normalization on the performance of edgeR, DESeq2, and limma-voom methods in simulated cell populations with $P = 11$ number of cell populations and 3 different sample sizes per group ($n = 5, 10$ and 20). The data is simulated using the non-parametric simulation procedure using the healthy samples from the Lupus dataset in processing cohort 4 as a baseline. Reported metrics are averages from 250 simulation runs for each simulation scenario. The reported performance metrics are at 1%, 5%, and 10% nominal FDR.

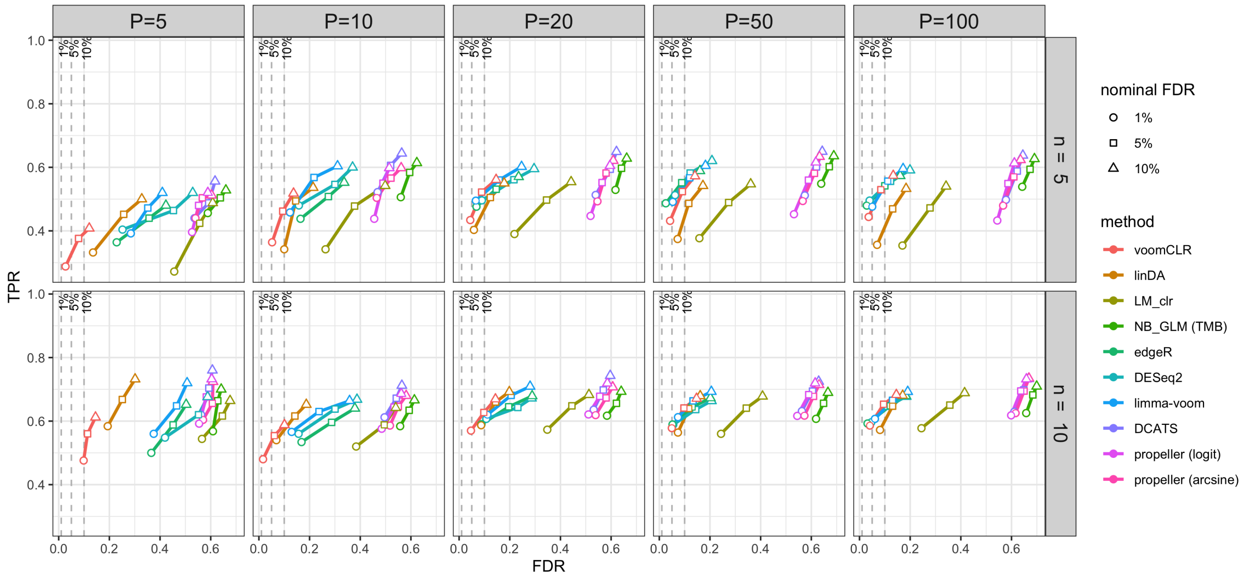

Supplementary Figure S12: *Simulation study:* FDR-TPR curves for evaluating the performance of voomCLR and other methods for 5 different numbers of cell populations ($P = 5, 10, 20, 50$ and 100). The data is simulated from a Dirichlet-Multinomial distribution, with medium level of variability (Dirichlet parameters scaling factor $\gamma = 1$), and 2 different sample sizes per group ($n = 5$ and 10). Reported metrics are averages from 250 simulation runs for each simulation scenario and performance metrics are calculated at 1%, 5%, and 10% nominal FDR.

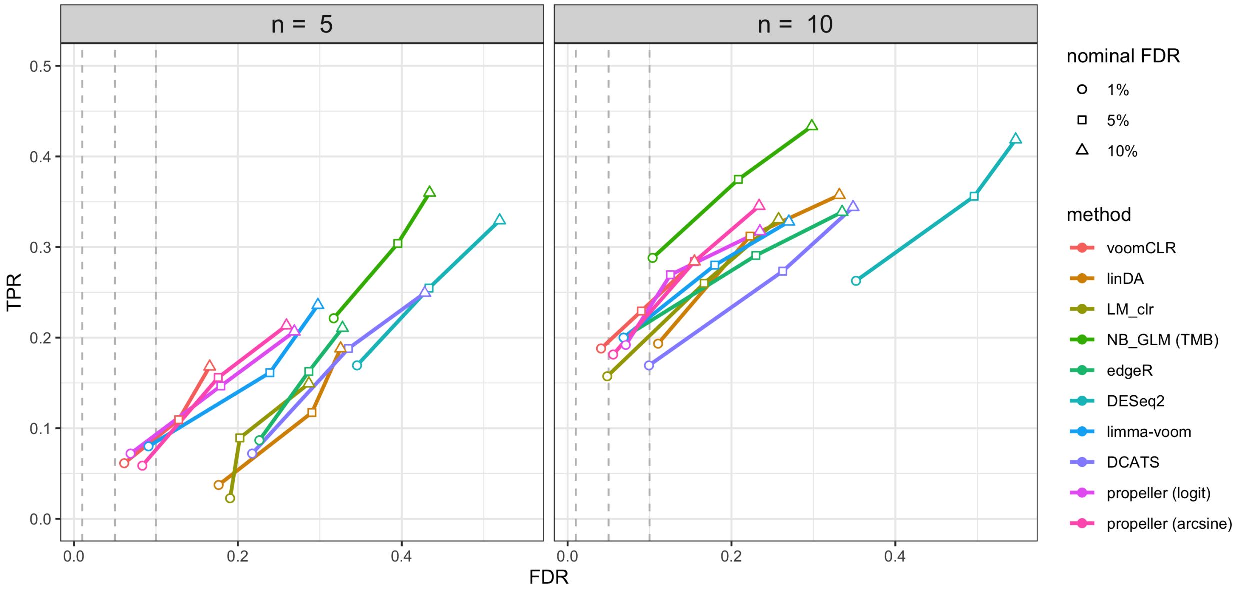

Supplementary Figure S13: *Simulation study:* FDR-TPR curves for evaluating the performance of voomCLR and other methods with non-parametrically simulated cell population count data using the Breast cell atlas data as input (healthy samples are used as the baseline for the simulation). The number of cell populations in the simulated data is 15. Reported metrics are averages from 250 simulation runs for each simulation scenario and performance metrics are calculated at 1%, 5%, and 10% nominal FDR

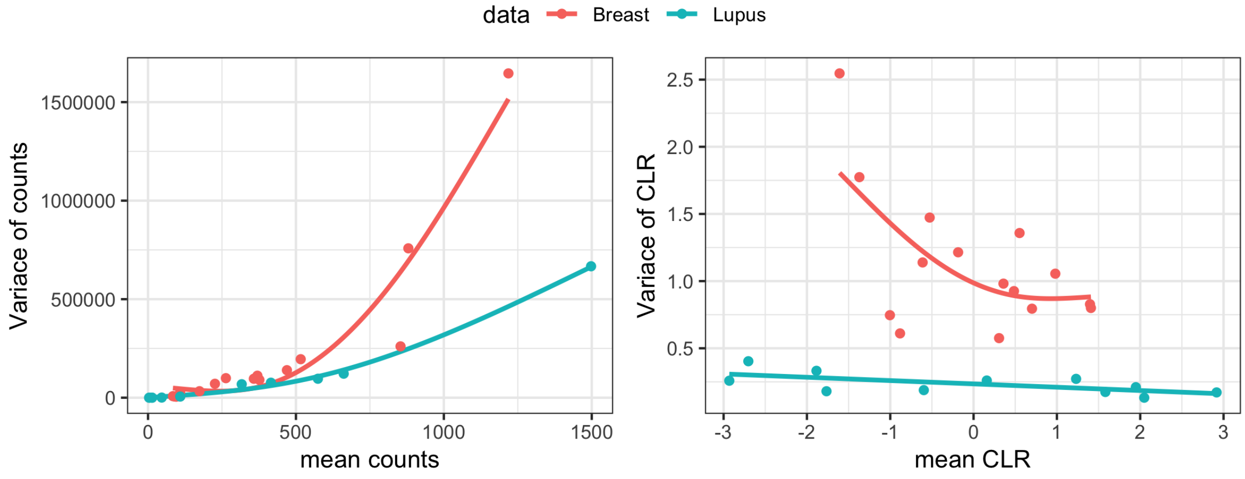

Supplementary Figure S14: *Breast atlas and Lupus case study: comparison of variability.* Mean-variance trend in the Breast atlas (healthy samples) and Lupus (healthy samples in processing cohort 4) datasets. Each point is a cell type and the solid lines are the smoothed trends for the mean variance association of counts (left) and CLR transformed counts (right).

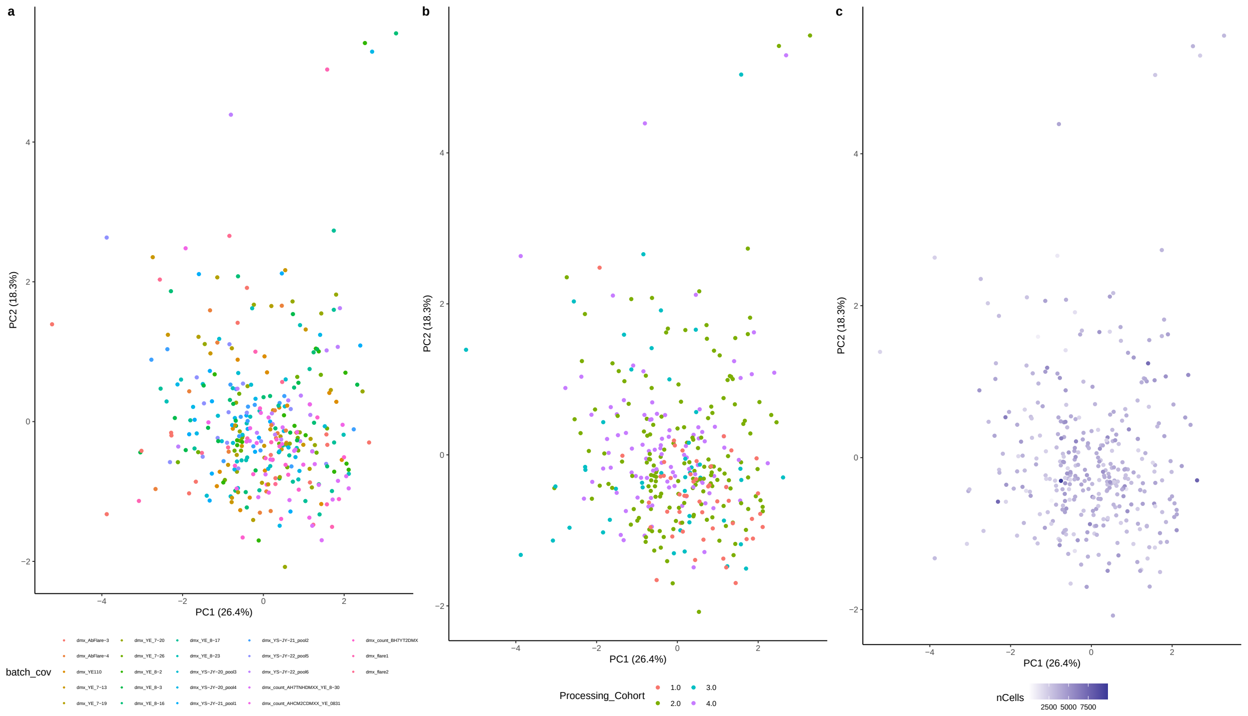

Supplementary Figure S15: *Lupus case study: Scatterplot of first two principal components of Aitchison's distance PCA, coloring by technical variables.* **(a)** Samples colored by batch. **(b)** Samples colored by processing cohort. **(c)** Samples colored by the total number of cells in each sample.

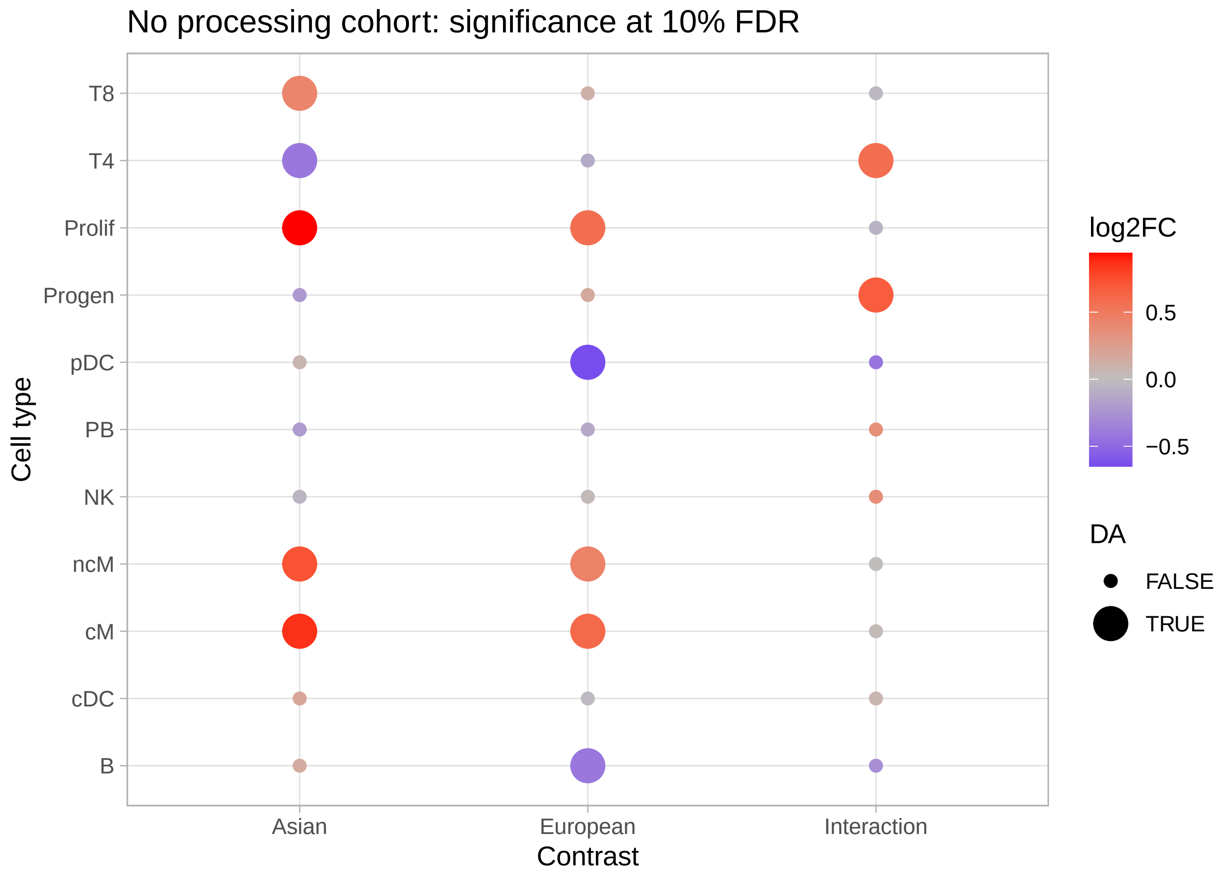

Supplementary Figure S16: *Lupus case study: Heatmap showing* voomCLR *results when not accounting for processing cohort.* Contrasts are denoted in columns and cell types in rows. Each point is colored according to the log2 fold-change, and point size denotes whether the FDR-adjusted p-value is below the 10% level.

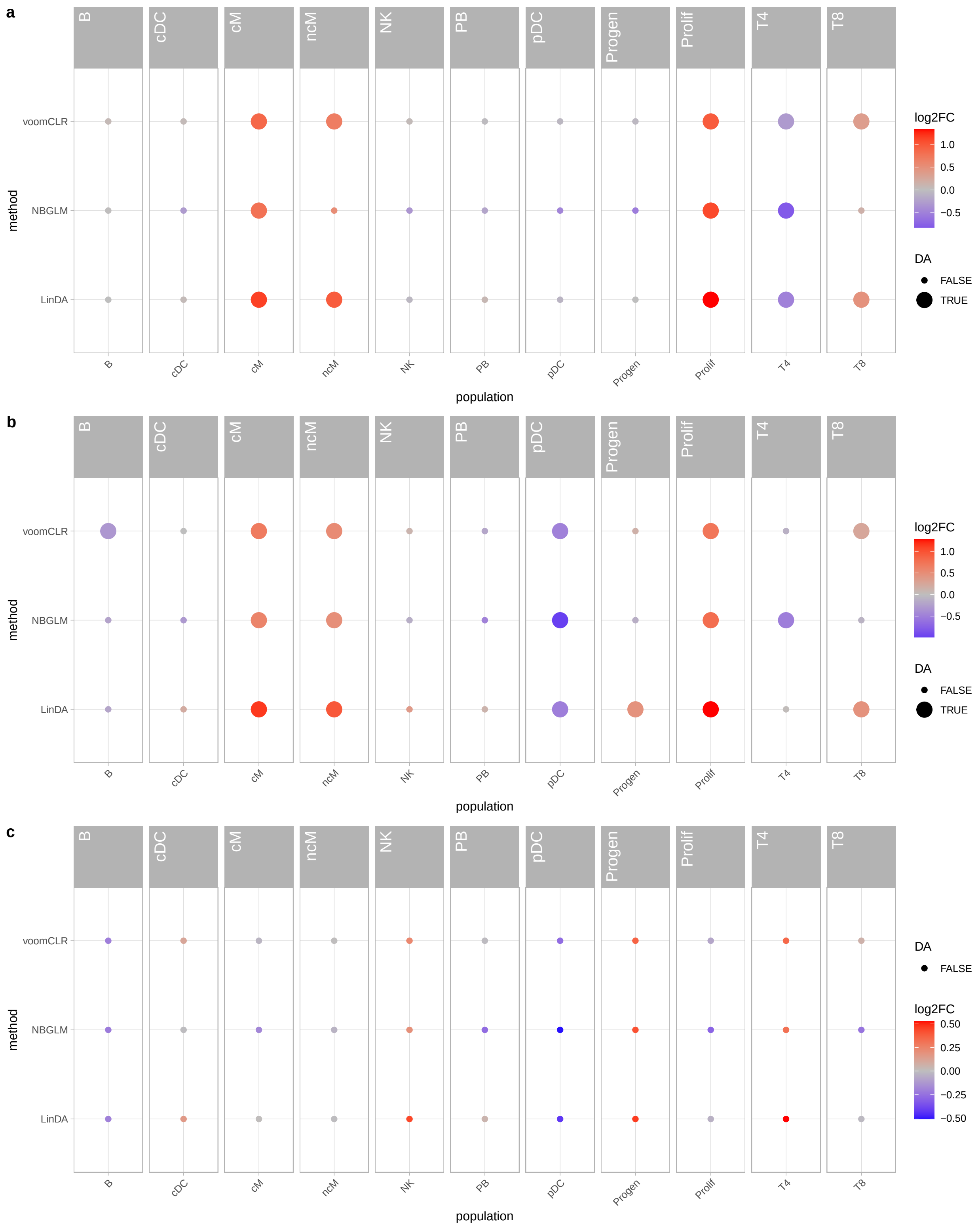

Supplementary Figure S17: *Lupus case study: Heatmaps showing results of three methods (rows) for all cell types (columns).* Each point is colored according to the log2 fold-change, and point size denotes whether the FDR-adjusted p-value is below the 10% level. **(a)** Results for comparing lupus versus healthy samples with Asian ancestry. **(b)** Results for comparing lupus versus healthy samples with European ancestry. **(c)** Inference results on the lupus disease $\times$ ancestry interaction effect.

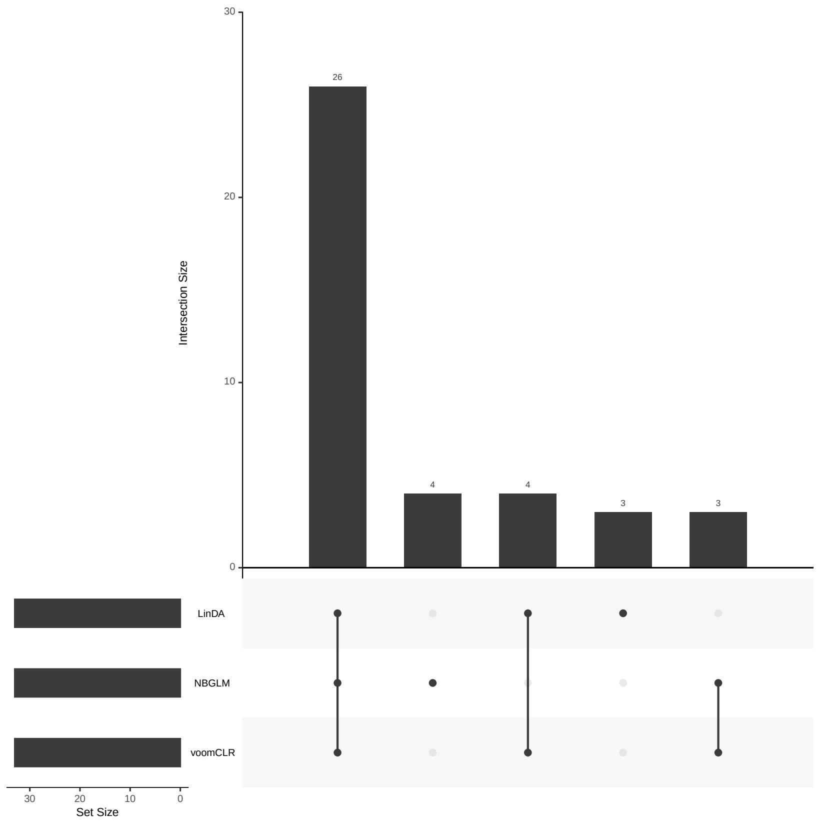

Supplementary Figure S18: *Lupus case study: Upset plot for lupus case study.*

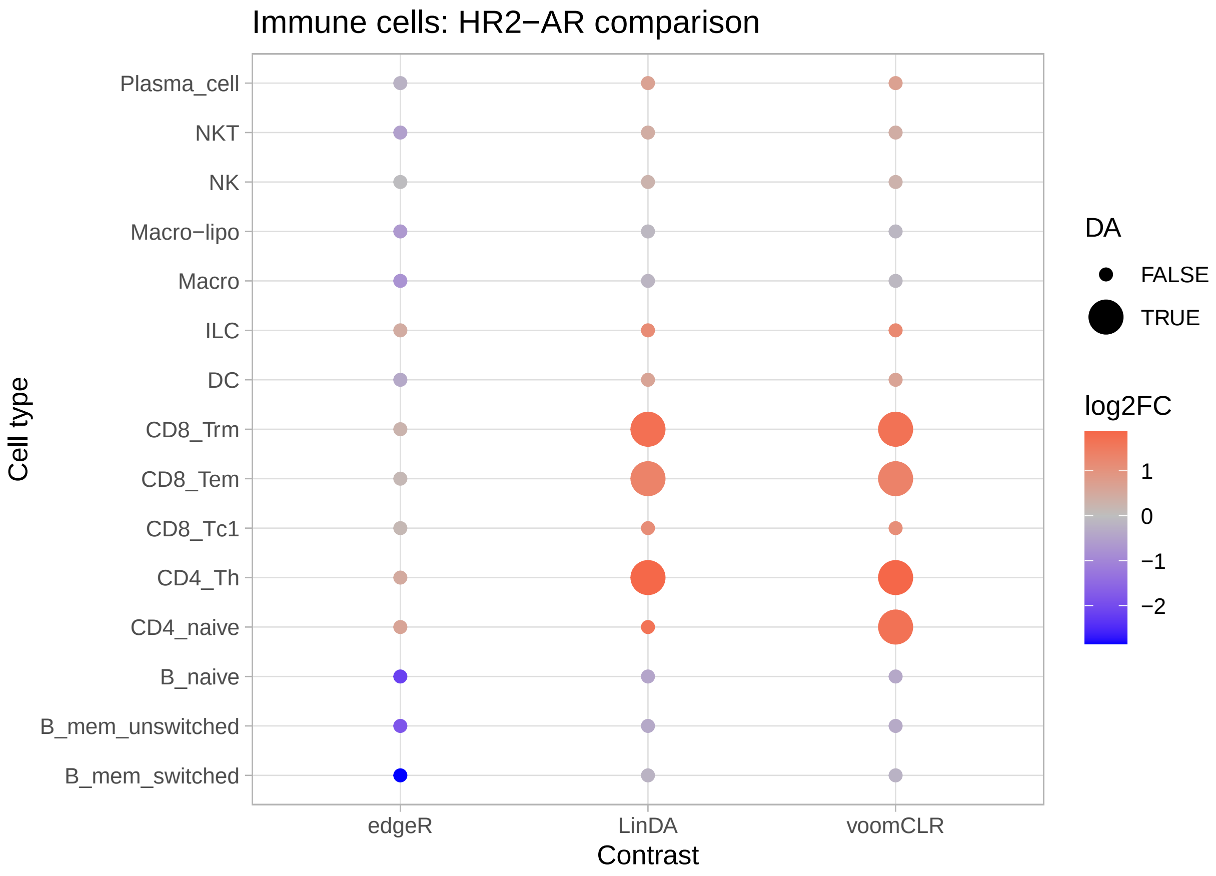

Supplementary Figure S19: *Breast atlas case study: HR2 versus AR donors comparison.* Heatmap showing results of edgeR, LinDA and voomCLR without bootstrapping, for all cell types (y-axis) in the comparison of HR-BR2 versus AR donors. Each point is colored according to the log2 fold-change (positive is higher abundance in HR-BR2 donors), and point size denotes whether the FDR-adjusted p-value is below the 5% level.

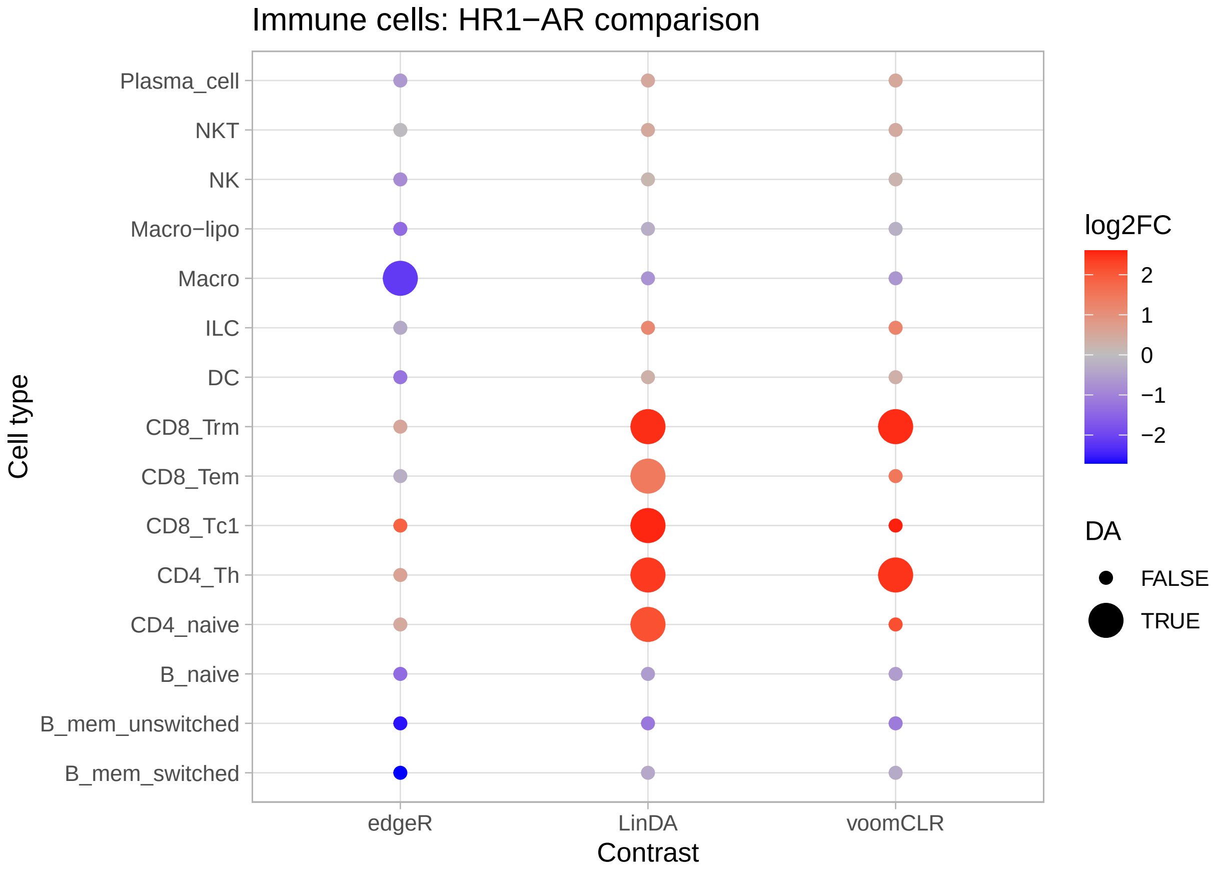

Supplementary Figure S20: *Breast atlas case study: HR1 versus AR donors comparison, using the parametric bootstrap for voomCLR.* Heatmap showing results of edgeR, LinDA and voomCLR with parametric bootstrapping, for all cell types (y-axis) in the comparison of HR-BR1 versus AR donors. Each point is colored according to the log2 fold-change (positive is higher abundance in HR-BR1 donors), and point size denotes whether the FDR-adjusted p-value is below the 5% level.

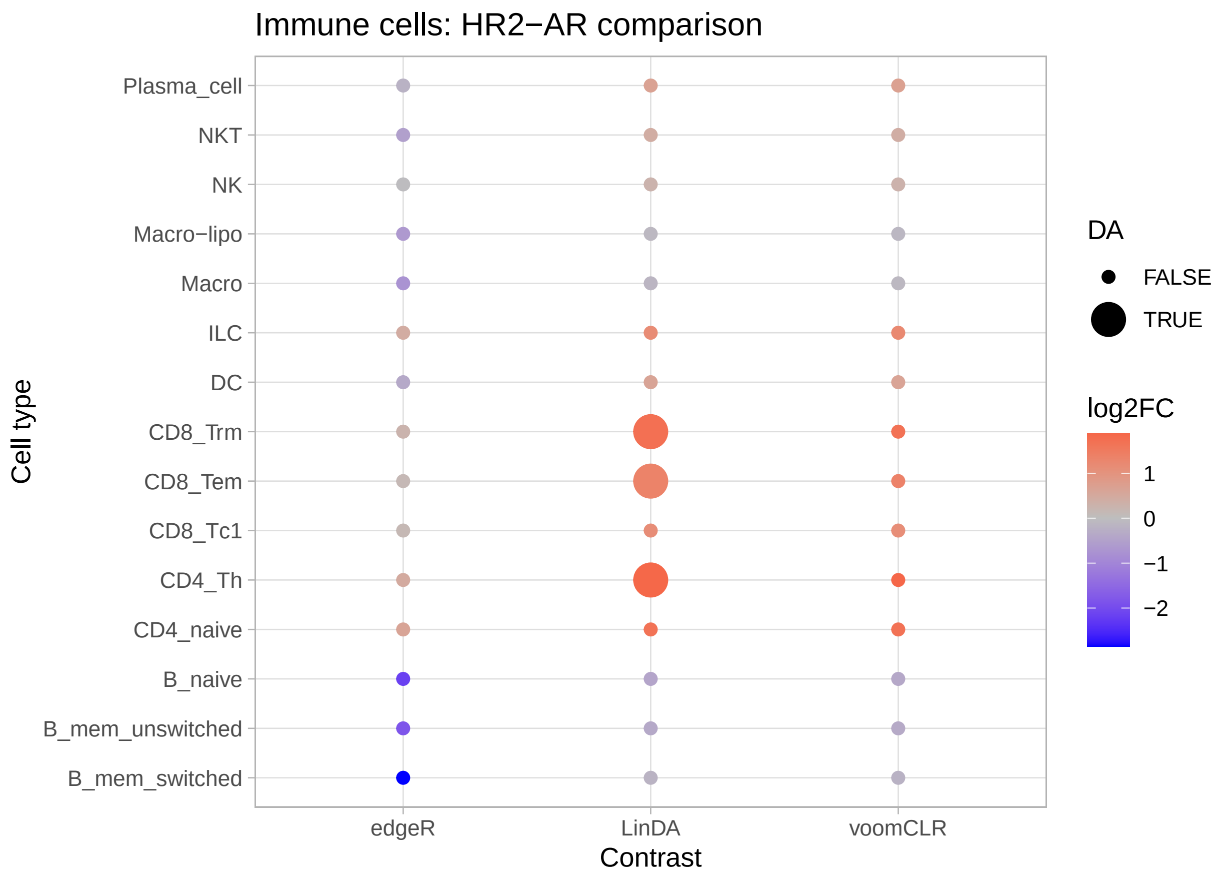

Supplementary Figure S21: *Breast atlas case study: HR2 versus AR donors comparison, using the parametric bootstrap for voomCLR.* Heatmap showing results of edgeR, LinDA and voomCLR with parametric bootstrapping, for all cell types (y-axis) in the comparison of HR-BR2 versus AR donors. Each point is colored according to the log2 fold-change (positive is higher abundance in HR-BR2 donors), and point size denotes whether the FDR-adjusted p-value is below the 5% level.

References

Mollie E. Brooks, Kasper Kristensen, Koen J. van Benthem, Arni Magnusson, Casper W. Berg, Anders Nielsen, Hans J. Skaug, Martin Mächler, and Benjamin M. Bolker. glmmTMB Balances Speed and Flexibility Among Packages for Zero-inflated Generalized Linear Mixed Modeling. *The R Journal*, 9(2):378–400, 2017. ISSN 2073-4859. URL https://journal.r-project.org/archive/2017/RJ-2017-066/index.html.

Emma Dann, Neil C. Henderson, Sarah A. Teichmann, Michael D. Morgan, and John C. Marioni. Differential abundance testing on single-cell data using k-nearest neighbor graphs. *Nature Biotechnology*, 40(2):245–253, February 2022. ISSN 1546-1696. doi: 10.1038/s41587-021-01033-z. URL https://www.nature.com/articles/s41587-021-01033-z. Number: 2 Publisher: Nature Publishing Group.

J. L. Doob. The Limiting Distributions of Certain Statistics. *The Annals of Mathematical Statistics*, 6(3):160–169, September 1935. ISSN 0003-4851, 2168-8990. doi: 10.1214/aoms/1177732594. URL https://projecteuclid.org/journals/annals-of-mathematical-statistics/volume-6/issue-3/The-Limiting-Dis Publisher: Institute of Mathematical Statistics.

Robert Dorfman. A note on the Delta-Method for finding Variance Formulae. *The Biometric Bulletin*, 1(1): 129–137, 1938.

Charity W. Law, Yunshun Chen, Wei Shi, and Gordon K. Smyth. voom: precision weights unlock linear model analysis tools for RNA-seq read counts. *Genome Biology*, 15(2):R29, February 2014. ISSN 1474-760X. doi: 10.1186/gb-2014-15-2-r29. URL https://doi.org/10.1186/gb-2014-15-2-r29.

Xinyi Lin, Chuen Chau, Kun Ma, Yuanhua Huang, and Joshua W. K. Ho. DCATS: differential composition analysis for flexible single-cell experimental designs. *Genome Biology*, 24(1):151, June 2023. ISSN 1474-760X. doi: 10.1186/s13059-023-02980-3. URL https://doi.org/10.1186/s13059-023-02980-3.

Michael I. Love, Wolfgang Huber, and Simon Anders. Moderated estimation of fold change and dispersion for RNA-seq data with DESeq2. *Genome Biology*, 15(12):550, December 2014. ISSN 1474-760X. doi: 10.1186/s13059-014-0550-8. URL https://doi.org/10.1186/s13059-014-0550-8.

Stefano Mangiola, Alexandra J. Roth-Schulze, Marie Trussart, Enrique Zozaya-Vald©s, Mengyao Ma, Zijie Gao, Alan F. Rubin, Terence P. Speed, Heejung Shim, and Anthony T. Papenfuss. sccomp: Robust differential composition and variability analysis for single-cell data. *Proceedings of the National Academy of Sciences*, 120(33):e2203828120, 2023. doi: 10.1073/pnas.2203828120. URL https://www.pnas.org/doi/abs/10.1073/pnas.2203828120.

Richard K. Perez, M. Grace Gordon, Meena Subramaniam, Min Cheol Kim, George C. Hartoularos, Sasha Targ, Yang Sun, Anton Ogorodnikov, Raymund Bueno, Andrew Lu, Mike Thompson, Nadav Rappoport, Andrew Dahl, Cristina M. Lanata, Mehrdad Matloubian, Lenka Maliskova, Serena S. Kwek, Tony Li, Michal Slyper, Julia Waldman, Danielle Dionne, Orit Rozenblatt-Rosen, Lawrence Fong, Maria Dall'Era, Brunilda Balliu, Aviv Regev, Jinoos Yazdany, Lindsey A. Criswell, Noah Zaitlen, and Chun Jimmie Ye. Single-cell RNA-seq reveals cell type-specific molecular and genetic associations to lupus. *Science (New York, N.Y.)*, 376(6589):eabf1970, April 2022. ISSN 1095-9203. doi: 10.1126/science.abf1970.

Belinda Phipson, Choon Boon Sim, Enzo R Porrello, Alex W Hewitt, Joseph Powell, and Alicia Oshlack. propeller: testing for differences in cell type proportions in single cell data. *Bioinformatics*, 38(20):4720–4726, October 2022. ISSN 1367-4803. doi: 10.1093/bioinformatics/btac582. URL https://doi.org/10.1093/bioinformatics/btac582.

Austin D. Reed, Sara Pensa, Adi Steif, Jack Stenning, Daniel J. Kunz, Linsey J. Porter, Kui Hua, Peng He, Alecia-Jane Twigger, Abigail J. Q. Siu, Katarzyna Kania, Rachel Barrow-McGee, Iain Goulding, Jennifer J. Gomm, Valerie Speirs, J. Louise Jones, John C. Marioni, and Walid T. Khaled. A single-cell atlas enables mapping of homeostatic cellular shifts in the adult human breast. *Nature Genetics*, 56(4):652–662, April 2024. ISSN 1546-1718. doi: 10.1038/s41588-024-01688-9. URL

https://www.nature.com/articles/s41588-024-01688-9. Number: 4 Publisher: Nature Publishing Group.

Mark D. Robinson and Alicia Oshlack. A scaling normalization method for differential expression analysis of RNA-seq data. *Genome Biology*, 11(3):R25, March 2010. ISSN 1474-760X. doi: 10.1186/gb-2010-11-3-r25. URL https://doi.org/10.1186/gb-2010-11-3-r25.

Mark D. Robinson, Davis J. McCarthy, and Gordon K. Smyth. edgeR: a Bioconductor package for differential expression analysis of digital gene expression data. *Bioinformatics*, 26 (1):139–140, January 2010. ISSN 1367-4803. doi: 10.1093/bioinformatics/btp616. URL https://doi.org/10.1093/bioinformatics/btp616.

Gordon K. Smyth. Linear Models and Empirical Bayes Methods for Assessing Differential Expression in Microarray Experiments. *Statistical Applications in Genetics and Molecular Biology*, 3(1), February 2004. ISSN 1544-6115. doi: 10.2202/1544-6115.1027. URL https://www.degruyter.com/document/doi/10.2202/1544-6115.1027/html. Publisher: De Gruyter.

Charlotte Soneson and Mark D. Robinson. iCOBRA: open, reproducible, standardized and live method benchmarking. *Nature Methods*, 13(4):283–283, April 2016. ISSN 1548-7105. doi: 10.1038/nmeth.3805. URL https://www.nature.com/articles/nmeth.3805. Publisher: Nature Publishing Group.
